# Deficiency of ADAR2 ameliorates metabolic-associated fatty liver disease via AMPK signaling pathways in obese mice

**DOI:** 10.1038/s42003-024-06215-4

**Published:** 2024-05-17

**Authors:** Mei-Lang Kung, Siao Muk Cheng, Yun-Han Wang, Kai-Pi Cheng, Yu-Lin Li, Yi-Tsen Hsiao, Bertrand Chin-Ming Tan, Yun-Wen Chen

**Affiliations:** 1 Department of Medical Education and Research, Kaohsiung Veterans General Hospital, Kaohsiung, Taiwan; 2 National Institute of Cancer Research, National Health Research Institutes (NHRI), Tainan, Taiwan; 3 Department of Pharmacology, College of Medicine, National Cheng Kung University, Tainan, Taiwan; 4 Department of Internal Medicine, National Cheng Kung University Hospital, College of Medicine, National Cheng Kung University, Tainan, Taiwan; 5 Graduate Institute of Biomedical Sciences, College of Medicine, Chang Gung University, Taoyuan, Taiwan; 6 Department of Biomedical Sciences, College of Medicine, Chang Gung University, Taoyuan, Taiwan; 7 Department of Neurosurgery, Linkou Medical Center, Chang Gung Memorial Hospital, Linkou, Taiwan; 8 Research Center for Emerging Viral Infections, Chang Gung University, Taoyuan, Taiwan

**Keywords:** Metabolic syndrome, Metabolic disorders

## Abstract

Non-alcoholic fatty liver disease (NAFLD) is a chronic disease caused by hepatic steatosis. Adenosine deaminases acting on RNA (ADARs) catalyze adenosine to inosine RNA editing. However, the functional role of ADAR2 in NAFLD is unclear. ADAR2^+/+^/GluR-B^R/R^ mice (wild type, WT) and ADAR2^−/−^/GluR-B^R/R^ mice (ADAR2 KO) mice are fed with standard chow or high-fat diet (HFD) for 12 weeks. ADAR2 KO mice exhibit protection against HFD–induced glucose intolerance, insulin resistance, and dyslipidemia. Moreover, ADAR2 KO mice display reduced liver lipid droplets in concert with decreased hepatic TG content, improved hepatic insulin signaling, better pyruvate tolerance, and increased glycogen synthesis. Mechanistically, ADAR2 KO effectively mitigates excessive lipid production via AMPK/Sirt1 pathway. ADAR2 KO inhibits hepatic gluconeogenesis via the AMPK/CREB pathway and promotes glycogen synthesis by activating the AMPK/GSK3β pathway. These results provide evidence that ADAR2 KO protects against NAFLD progression through the activation of AMPK signaling pathways.

## Abstract (web summary)

ADAR2 KO mice are protected against several HFD–induced metabolic defects, show reduced hepatic lipid accumulation, improved hepatic insulin signaling, and increased pyruvate tolerance and glycogen synthesis, by activation of AMPK signaling pathways.

## Introduction

Non-alcoholic fatty liver disease (NAFLD) is the most common form of chronic liver disease worldwide. The prevalence of NAFLD in the general population of Western countries is from 17% to 46% and is rapidly increasing in parallel with the increasing prevalence of obesity and metabolic syndrome^1^. NAFLD is a complex multifaceted disease frequently correlated with metabolic comorbidities, including type 2 diabetes mellitus (T2DM), dyslipidemia, obesity, and hyperlipidemia^2^. The global prevalence of NAFLD ranges from 50% to 70% among T2DM patients^3,4^. NAFLD encompasses a broad spectrum of pathological conditions ranging from simple steatosis (NAFL) and nonalcoholic steatohepatitis (NASH) to cirrhosis and hepatocellular carcinoma^5^. The crucial feature of NAFLD is an excess of fatty acids in the liver, which leads to triglycerides (TG) accumulation (steatosis). Hepatic fat accumulation results from an imbalance between lipid acquisition and disposal, regulated via four major pathways, including hepatic lipid uptake, de novo lipogenesis, fatty acids oxidation, and lipid export^6^. De novo lipogenesis derived from a high-fat diet is a key process for lipid synthesis in the liver, which is primarily regulated by sterol regulatory element-binding protein-1c (SREBP-1c)^7^. Elevated expression of SREBP-1c results in the transcriptional activation of downstream crucial lipogenic rate‐limiting enzymes involved in de novo lipogenesis, including acetyl-CoA carboxylase 1 (ACC1), fatty acid synthase (FAS), ATP‐citrate lyase (ACL) and stearoyl‐CoA desaturase‐1 (SCD1)^8^. Studies have shown that increased expression of SREBP-1c was observed in patients with NAFLD^9^.

Adenosine-to-inosine (A-to-I) RNA editing, which is the process catalyzed by a highly conserved family known as adenosine deaminase acting on RNA (ADARs), is a ubiquitous and important post-transcriptional modification of genome-encoded RNA transcripts^10,11^. Three fundamentally related members of the ADAR family have been identified in the vertebrate genome: ADAR1 and ADAR2, which are commonly expressed in many tissues, and ADAR3, which is specifically expressed in neuronal tissue and is believed to be catalytically inactive^12–14^. Abnormal ADAR expression or disrupted A-to-I RNA editing has been closely related to many human diseases, including cancer, neurological disorders, metabolic disease, viral infection, and autoimmune disorders^15–19^. Studies showed that the expression level of ADAR2 was upregulated in pancreatic islets of HFD-induced obese mice^19,20^. Knockdown of ADAR2 reduced glucose-stimulated insulin secretion in rat INS-1 cells and pancreatic islets^18^. ADAR2-mediated 5-HT2cR editing impaired glucose-stimulated insulin secretion (GSIS) via altered endoplasmic reticulum (ER) calcium release and impaired store-operated calcium entry (SOCE) activation in pancreatic MIN6 β cells^19^. Cui et al. showed that heterozygous knockout of ADAR1 protected against HFD-induced obesity and insulin resistance in mice^21^. A recent study done by Xiang et al. showed that ADAR1 overexpression ameliorates HFD-induced liver injury by inhibiting NLRP3 inflammasome^22^. Transgenic mice expressing either wild-type or inactive ADAR2 isoforms displayed adult-onset obesity characterized by hyperglycemia, hyperleptinemia, and increased adiposity^23^. However, the functional role of ADAR2 in hepatic steatosis, particularly in NAFLD, remains unclear.

In the present study, we established a mouse model of NAFLD by adopting a high-fat diet containing 60% kcal fat and aimed to determine the impact of ADAR2 deletion on hepatic lipid metabolism, insulin action, and whole-body glucose homeostasis. Herein, we showed that ADAR2 KO effectively prevented HFD-induced NAFLD progression by regulating glucose metabolism dysregulation, insulin resistance, and lipid accumulation, at least partially, through regulating AMP-activated protein kinase (AMPK)-mediated signaling cascade.

## Results

### ADAR2 KO suppresses lipid accumulation in hepatocytes

**Fig. 1:**
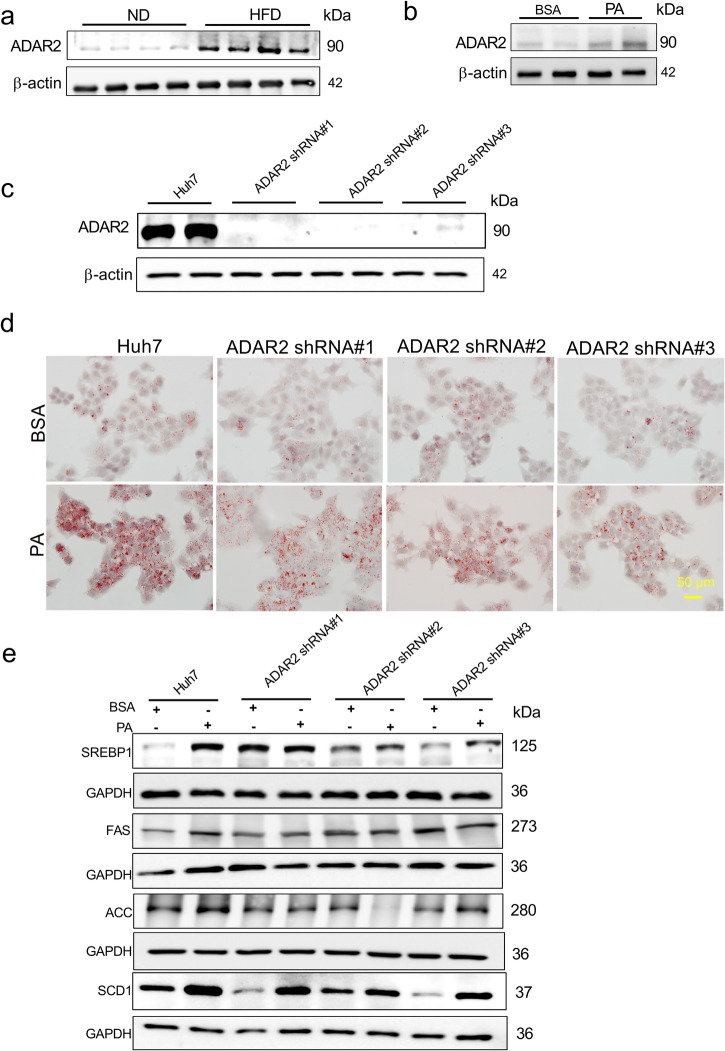
ADAR2 KO suppresses lipid accumulation in hepatocytes. **a** Western blot of ADAR2 expression in the livers of C57BL/6J mice after normal diet (ND) or high‐fat diet (HFD) consumption for 12 weeks (*n* = 4 mice/group). **b** Western blot of ADAR2 expression in Huh7 cells stimulated with palmitic acid (PA) (0.25 mM) for 24 h (*n* = 6, three independent experiments). **c** Western blot of ADAR2 expression in ADAR2-deficient Huh7 cells (*n* = 6, three independent experiments). **d** Representative Oil red O image of lipid drop accumulation in ADAR2-deficient Huh7 cells stimulated with palmitic acid (PA) (0.25 mM) for 24 h (*n* = 3, three independent experiments). **e** Western blot of de novo lipogenesis-related protein in ADAR2-deficient Huh7 cells stimulated with palmitic acid (PA) (0.25 mM) for 24 h (*n* = 3, three independent experiments).

To explore the relationship between ADAR2 and NAFLD, we examined the expression of ADAR2 in mice with HFD-induced fatty liver. Western blot analysis clearly showed that the protein expression of ADAR2 in the mouse liver was consistently markedly increased (Fig. 1a). A similar result was obtained in human hepatocyte cell line Huh7 treated with palmitic acid (PA) for 24 h (Fig. 1b). These data suggest that ADAR2 plays an essential role in the progression of NAFLD.

Next, the function of ADAR2 in NAFLD was studied by knockdown approaches. ADAR2 knockdown by different shRNA duplexes in human hepatocyte cell line Huh7 showed that the expression of ADAR2 was significantly suppressed (Fig. 1c). After stimulation with PA, the lipid droplet in ADAR2-deficient Huh7 cells were much less than those in control cells (Fig. 1d). Consistent with this finding, the expression of lipid-related genes (SREBP1, FAS, ACC, and SCD1) was significantly down-regulated in ADAR2-deficient Huh7 cells with PA stimulation compared with control cells (Fig. 1e). Taken together, these data revealed that ADAR2 KO could prevent PA-induced lipid accumulation in hepatocytes.

### ADAR2 KO prevents HFD-induced weight gain in both genders

**Fig. 2:**
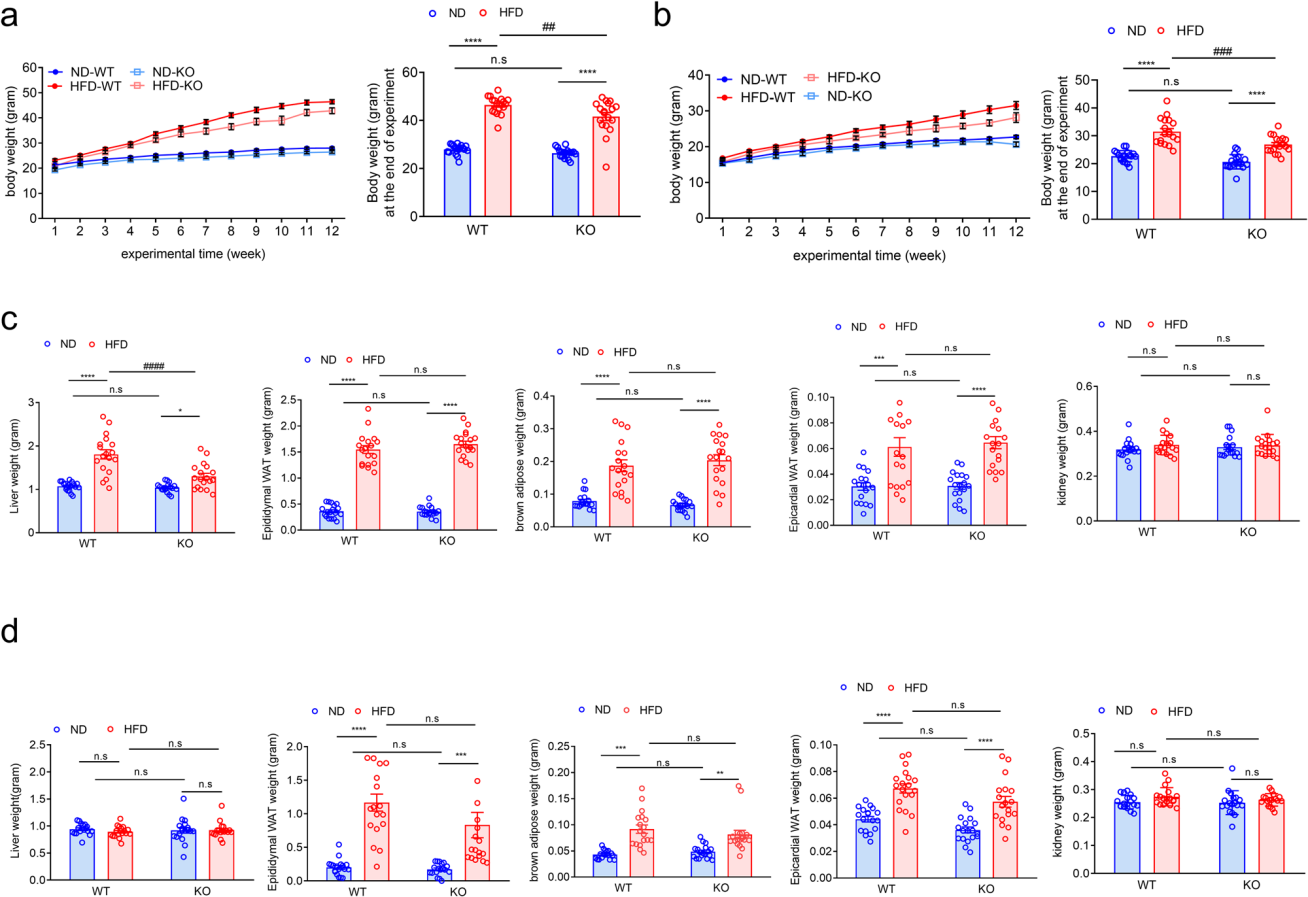
ADAR2 KO decreased body weight and liver weight in male obese mice. Physiological parameters in mice from the age of 5–17 weeks (*n* = 18 mice/group) (**a**, male; **b**, female) body weight (BW, g); (**c**, male; **d**, female) weights of liver, epididymal adipose, BAT, epicardial adipose, and kidney derived from WT and ADAR2 KO mice fed with ND or HFD are shown (*n* = 18) All data are expressed as mean ± SEM. Tukey’s multiple comparison test after the two-way ANOVA was conducted for (**a**–**d**). *ND-WT group versus HFD-WT group or ND-KO group versus HFD-KO group; **p < 0.01, ***p < 0.001, ****p < 0.0001; #HFD-WT group versus HFD-KO group; ##*p* < 0.01, ###*p* < 0.001, ####*p* < 0.0001; n.s, not significant.

To investigate the function of ADAR2 in hepatic steatosis in vivo, we used ADAR2^−/−^/ GluR-B^R/R^ mice that carry the edited (mutant) version of GluR, which rescues the early post-natal death of mice deficient in the RNA-editing enzyme ADAR2^24^. For simplicity reasons, henceforward, we refer to this strain as ADAR2^−/−^. ADAR2^+/+^ (Wild type, WT) and ADAR2^−/−^ (ADAR2 KO) mice were subjected to feed normal diet (ND) or HFD for 12 weeks. We first analyzed the body weight, energy intake, and water intake of both male and female mice fed with ND or HFD from the age of 5 weeks until 17 weeks. As expected, HFD progressively increased body weight (BW) in both genders of WT vs. ADAR2 KO mice (Fig. 2a, male; Fig. 2b, female). ADAR2 KO and WT mice on ND had similar body weight gain at 17 weeks. Notably, in HFD conditions, the body weight of ADAR2 KO mice was lower than that of WT mice in both genders (Fig. 2a, male; Fig. 2b, female). Energy intake and water intake showed no significant differences between each group in both genders (male, Supplementary Fig. 2a, b; Female, Supplementary Fig. 2c, d). We further found that oxygen consumption, carbon dioxide generation, respiratory exchange ratio as well as energy expenditure/heat generation showed no significant differences between male WT mice fed with HFD and male ADAR2 KO fed with HFD (Supplementary Fig. 3a–d). In agreement with this result, body fat and body lean composition showed no significant differences between male WT mice fed with HFD and male ADAR2 KO fed with HFD (Supplementary Fig. 3e, f).

### ADAR2 KO reduces liver tissue weight in male mice but not in female mice

Next, we characterized whether ADAR2 KO impacted the organ weight under HFD. As a result, liver weight was significantly lower in the male ADAR2 KO-HFD group compared with the male WT-HFD group (male, Fig. 2c). The liver weight showed no significant differences between each group in female mice (female, Fig. 2d). Both epicardial WAT, as well as epididymal WAT, BAT and kidney were not significantly different between ADAR2 KO-HFD mice and WT-HFD mice in both genders (male, Fig. 2c; Female, Fig. 2d).

### ADAR2 KO prevents male mice from HFD-induced glucose-metabolic dysfunctions

**Fig. 3:**
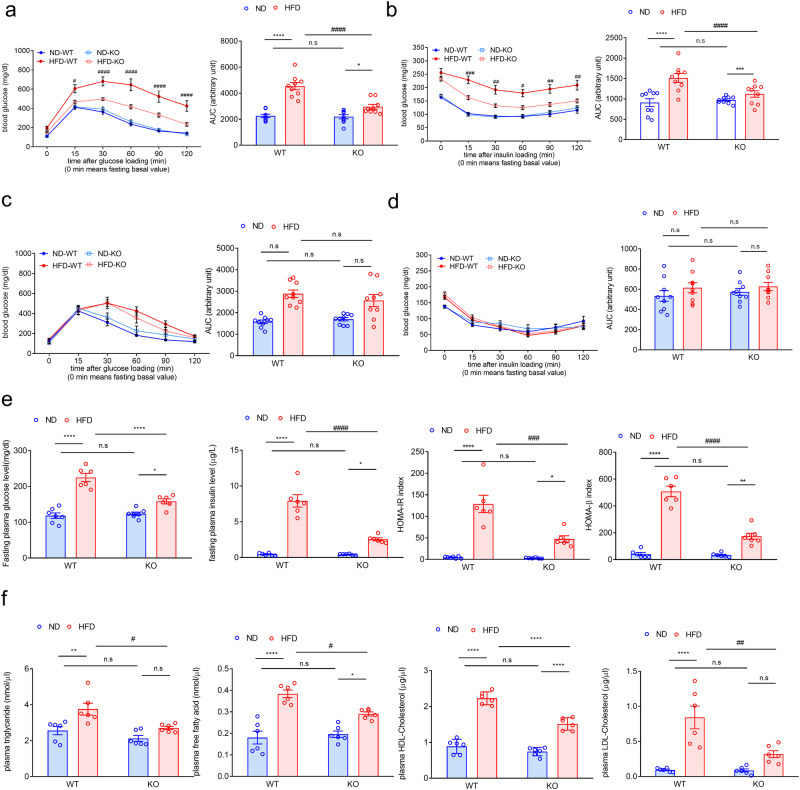
ADAR2 KO ameliorates glucose intolerance and insulin intolerance in male obese mice. The blood glucose levels in WT and ADAR2 KO mice fed with ND or HFD for 12 weeks (at the age of 17 weeks old) were measured at 2 h after glucose (male: **a**, female: **c**) and insulin (male: **b**, female: **d**) injections. ipGTT-area under the curve (AUC) quantification (0–120 min) (male: **a**, female: **c**) and ipITT-area under the curve (AUC) quantification (0–120 min) (male: **b**, female: **d**) were shown (*n* = 9 mice/group). **e** Fasting plasma glucose levels, fasting plasma insulin levels, and calculated HOMA-IR index of male mice (*n* = 6 mice/group). **f** Plasma levels of TG, free fatty acid, HDL-cholesterol, and LDL-cholesterol in male mice (*n* = 6 mice/group). All data are expressed as mean ± SEM. Tukey’s multiple comparison test after the two-way ANOVA was conducted for (**a**–**f**). *ND-WT group versus HFD-WT group or ND-KO group versus HFD-KO group; *p < 0.05, **p < 0.01, ***p < 0.001, ****p < 0.0001; #HFD-WT group versus HFD-KO group; #*p* < 0.05, ##*p* < 0.01, ###*p* < 0.001, ####*p* < 0.0001; n.s. not significant.

Because obesity impacts glucose metabolism and insulin action, we next analyzed the effects of ADAR2 deficiency on whole-body glucose utilization by performing a GTT on mice fed with ND or HFD for 12 weeks. After 12 weeks of HFD, ADAR2 KO mice displayed a better pattern of glucose tolerance (male, Fig. 3a) when compared with WT mice, but there is no difference in glucose tolerance between female WT and female ADAR2 KO of 12-week-HFD feeding (female, Fig. 3c). We then tested the insulin sensitivity of ADAR2 KO mice by performing an ITT. We found that male ADAR2 KO improved insulin sensitivity under HFD compared with WT mice (male, Fig. 3b). In contrast, those changes did not occur in female HFD mice (WT vs. ADAR2 KO) (female, Fig. 3d). These results showed that ADAR2 KO prevents male mice, but not female mice from HFD-induced glucose-metabolic dysfunctions. Therefore, we focused on male mice for the rest of the studies. Our plasma biochemical examinations showed elevated levels of plasma glucose, insulin, total cholesterol, free fatty acid, and triglycerides in the HFD obese mice (Fig. 3e, f). ADAR2 KO reversed these metabolic dysregulations (Fig. 3e, f). Moreover, the HOMA‐IR index was calculated to evaluate systemic insulin resistance. Compared with the ND group, the HOMA‐IR value was significantly increased with HFD treatment, whereas ADAR2 KO reversibly reduced the HOMA‐IR value when compared with that in WT‐treated HFD mice (Fig. 3e). The HOMA-β index was calculated to evaluate beta-cell function. Compared with the ND group, the HOMA‐β value was significantly increased with HFD treatment, whereas ADAR2 KO reversibly reduced the HOMA‐β value when compared with that in WT‐treated HFD mice (Fig. 3e).

### ADAR2 KO prevents male mice from HFD-induced hepatic lipid accumulation

**Fig. 4:**
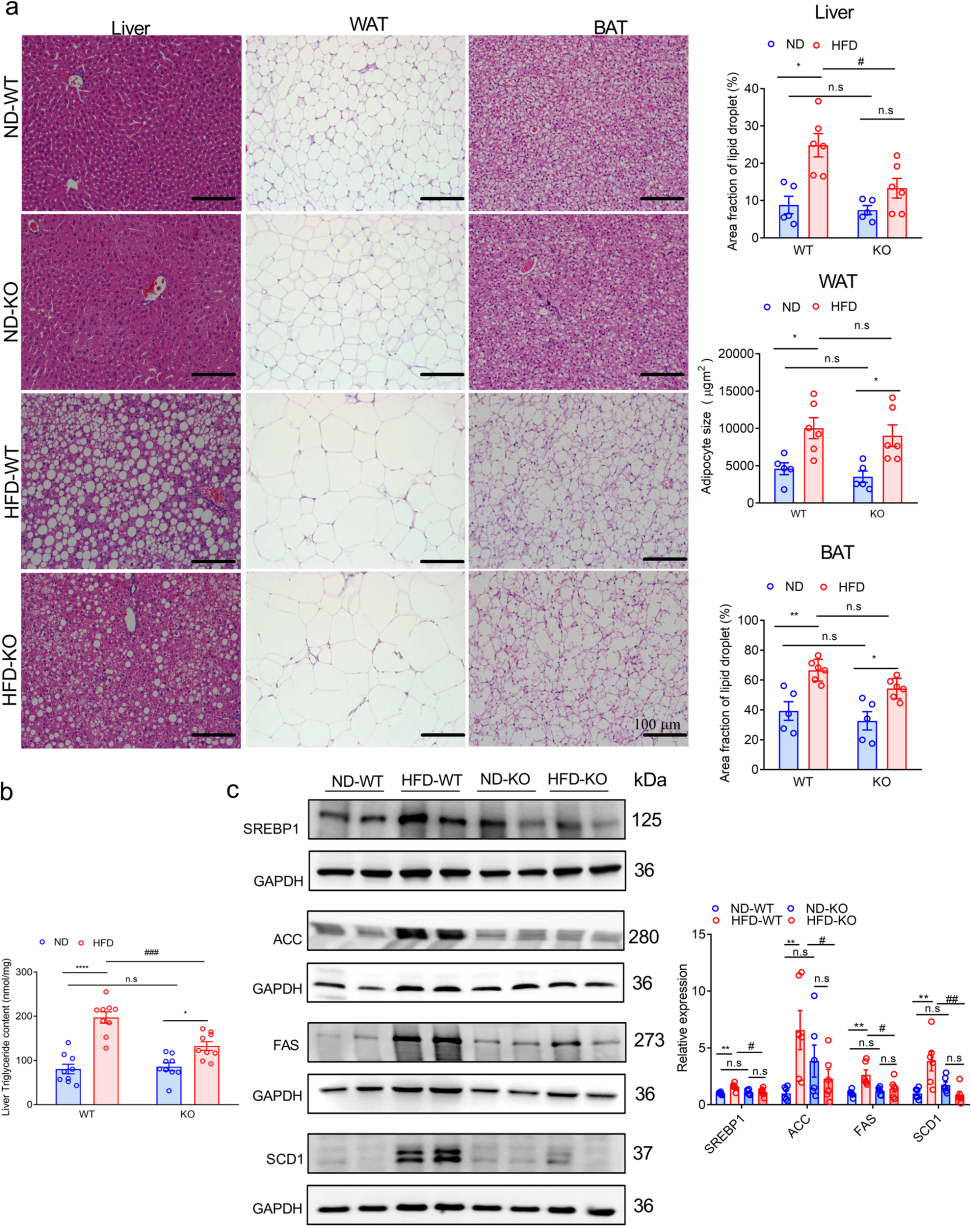
ADAR2 KO reduces lipid deposition in the liver. **a** Representative H&E staining of the liver, WAT, and BAT derived from WT and ADAR2 KO mice fed with ND or HFD is shown (Left panel), and quantitative analysis of lipid droplets of the liver, WAT and BAT in each group (Right panel). Scale bars = 100 µm (*n* = 5 mice/group). **b** Quantitative results of the liver TG content in each group (*n* = 9 mice/group). **c** Western blot assays of the proteins associated with the SREBP-1c-mediated lipogenesis pathway in each group (Left panel) and Quantitative analysis of the proteins associated with the SREBP-1c-mediated lipogenesis pathway in each group. All data were analyzed using 17-week-old mice after ND or HFD feeding for 12 weeks (*n* = 6 mice/group, three independent experiments). All data are expressed as mean ± SEM. Tukey’s multiple comparison test after the two-way ANOVA was conducted for (**a**–**c**). *ND-WT group versus HFD-WT group or ND-KO group versus HFD-KO group; *p < 0.05, **p < 0.01, ***p < 0.001, ****p < 0.0001; #HFD-WT group versus HFD-KO group; #*p* < 0.05, ##*p* < 0.01, ###*p* < 0.001; n.s, not significant.

As shown in Fig. 2c, male ADAR2 KO mice fed with HFD had lower liver weight when compared with male WT mice fed with HFD. Histological analyses further showed that ADAR2 KO mice fed with HFD (Fig. 4a), but not ND (Fig. 4a), had markedly reduced lipid deposition in the liver, but not in WAT and BAT. Hepatic TG content was reduced in ADAR2 KO mice fed with HFD compared with that of WT mice fed with HFD (Fig. 4b). Moreover, the expressions of SREBP-1c, ACC, FAS, and SCD1 were reduced in the liver of ADAR2 KO mice fed with HFD compared with that of WT mice fed with HFD (Fig. 4c). These results indicated that ADAR2 KO prevented liver against lipid accumulation through inhibiting de novo lipogenesis.

### Improved insulin signaling specifically in the liver of ADAR2 KO mice

**Fig. 5:**
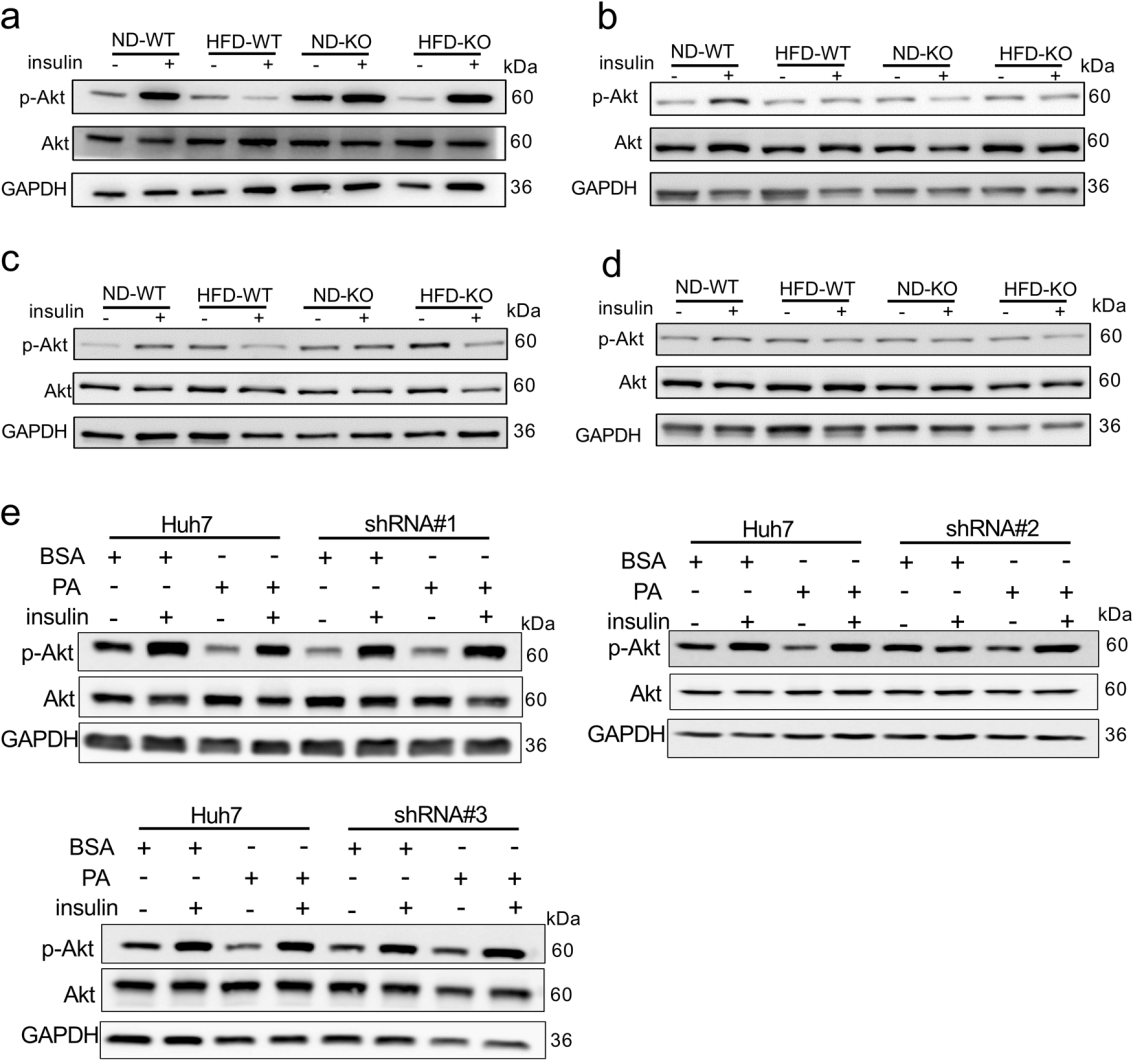
Insulin signaling in ADAR2 KO mice and ADAR2-deficient Huh7 cells. Immunoblot analyses on phosphorylation at Ser473 of Akt in the **a** liver, **b** muscle, **c** WAT, and **d** BAT of mice fed with ND and HFD. Each band represents a tissue extract from a single mouse. All data were analyzed using 17-week-old mice after ND or HFD feeding for 12 weeks (*n* = 3 mice/group, three independent experiments). **e** ADAR2-deficient Huh7 cells. Cells were treated with 0.25 mM PA for 24 h and then were stimulated with or without insulin for 30 min (*n* = 3 mice/group, three independent experiments).

As shown in Fig. 3, ADAR2 KO prevents male mice from HFD-induced systemic insulin resistance. To determine which insulin-responsive tissue(s) exhibited improved insulin signaling in ADAR2 KO mice, we examined the insulin action in mice fed with HFD. Insulin-stimulated phosphorylation at Ser473 of protein kinase B (Akt) was increased in liver (Fig. 5a), but not in the muscle (Fig. 5b), WAT (Fig. 5c) and BAT (Fig. 5d) of ADAR2 KO mice, when compared with those of WT mice (Fig. 5a–d). The results were confirmed in ADAR2-deficient Huh7 cells treated with PA for 24 h, suggesting that the knockdown of ADAR2 improved PA-impaired insulin signaling (Fig. 5e).

### Improved glucose metabolism in the liver of ADAR2 KO mice

**Fig. 6:**
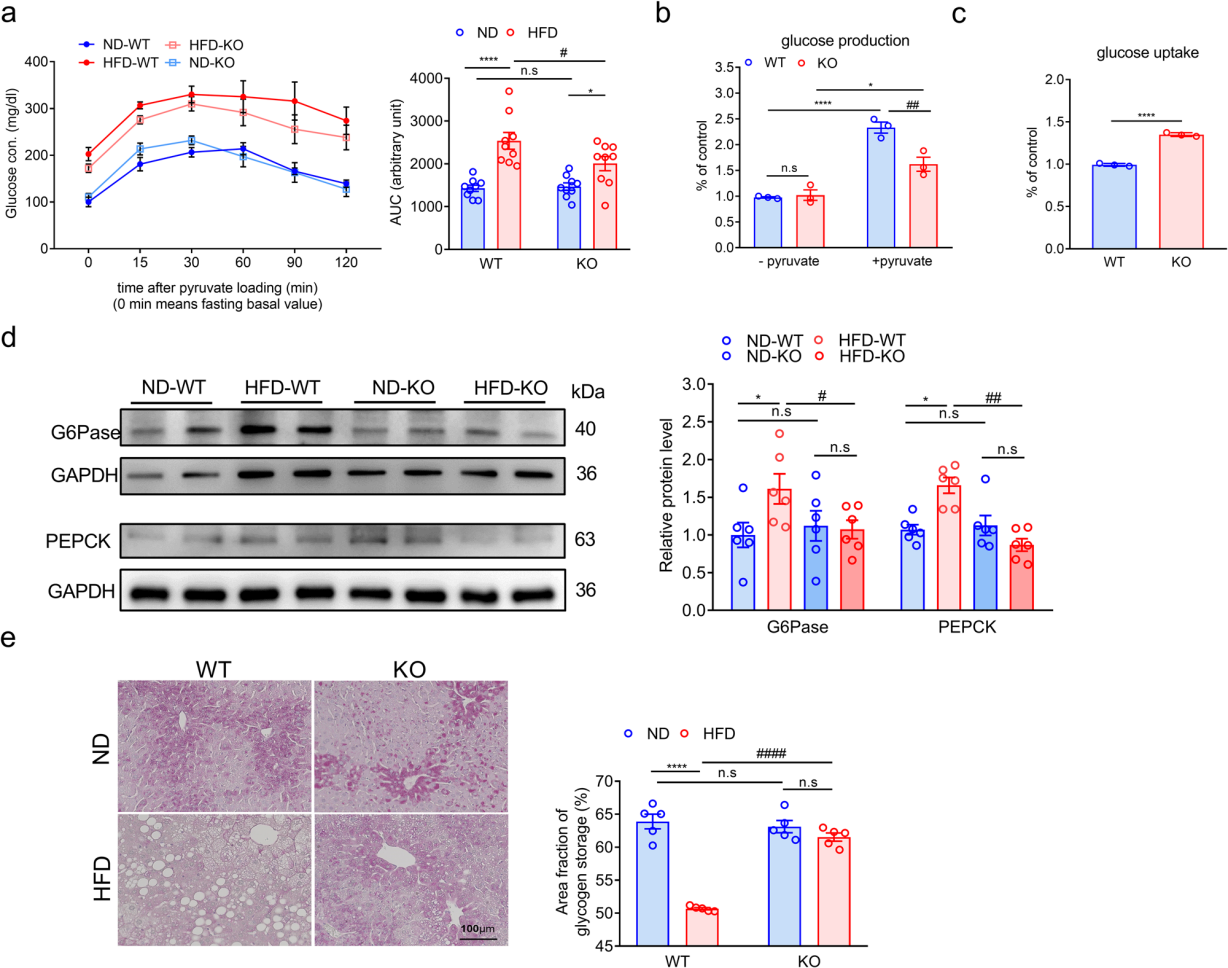
Glucose metabolism in the liver of ADAR2 KO mice. **a** Plasma glucose levels during PTT in male mice fed with ND and HFD and analysis of area under the curve (AUC) of IPPTT results. *n* = 9 mice per group. **b** Basal and pyruvate-induced glucose release into the medium after 4 h incubation in primary hepatocytes isolated from ADAR2 KO and WT mice. Results are derived from three independent experiments performed in triplicate. **c** Insulin-stimulated glucose uptake in primary hepatocytes from ADAR2 KO and WT mice. Results are derived from three independent experiments performed in triplicate. **d** Western blot assays of the proteins associated with gluconeogenesis in each group and Quantitative analysis of the proteins associated with gluconeogenesis in each group (*n* = 6 mice/group, three independent experiments). **e** Representative photographs of PAS staining in liver sections. *n* = 5 mice per group. All data are expressed as mean ± SEM. Tukey’s multiple comparison test after the two-way ANOVA was conducted for (**a**, **b**, **d** and **e**). Unpaired two-tailed Student’s *t*-test was conducted for (**c**). *ND-WT group versus HFD-WT group or ND-KO group versus HFD-KO group; *p < 0.05, ****p < 0.0001; #HFD-WT group versus HFD-KO group; #*p* < 0.05, ##*p* < 0.01, ####*p* < 0.0001; n.s, not significant.

To determine the functional correlates of increased insulin signaling in the livers of ADAR2 KO mice, we determined aspects of hepatic glucose metabolism. Pyruvate tolerance tests showed that glucose levels were significantly lower in ADAR2 KO mice fed with HFD in response to the administration of pyruvate, a major gluconeogenesis substrate (Fig. 6a). To further investigate the regulation of gluconeogenesis, we examined glucose production and gluconeogenic gene expression. ADAR2 deficiency significantly reduced pyruvate-induced glucose release into the medium by primary hepatocytes (Fig. 6b) and ADAR2-deficient Huh7 cells (Supplementary Fig. 4). Consistent with these observations, protein levels of phosphoenolpyruvate carboxykinase (PEPCK) and Glucose 6-phosphatase (G6Pase) in the liver of ADAR2 KO mice fed with HFD were significantly decreased compared with WT mice fed with HFD (Fig. 6d). To examine the functional effects of ADAR2 deficiency on hepatocyte insulin responsiveness, the ability of insulin to stimulate hepatocyte glucose uptake was determined. As expected, glucose uptake in isolated primary hepatocytes from ADAR2 KO mice was increased (Fig. 6c). Similar results were observed in ADAR2-deficient Huh7 cells (Supplementary Fig. 5). Moreover, PAS staining showed that the decreased glycogen in HFD mice was prevented by ADAR2 deficiency (Fig. 6e).

### ADAR2 KO activates the AMPK signaling cascade during NAFLD progression

**Fig. 7:**
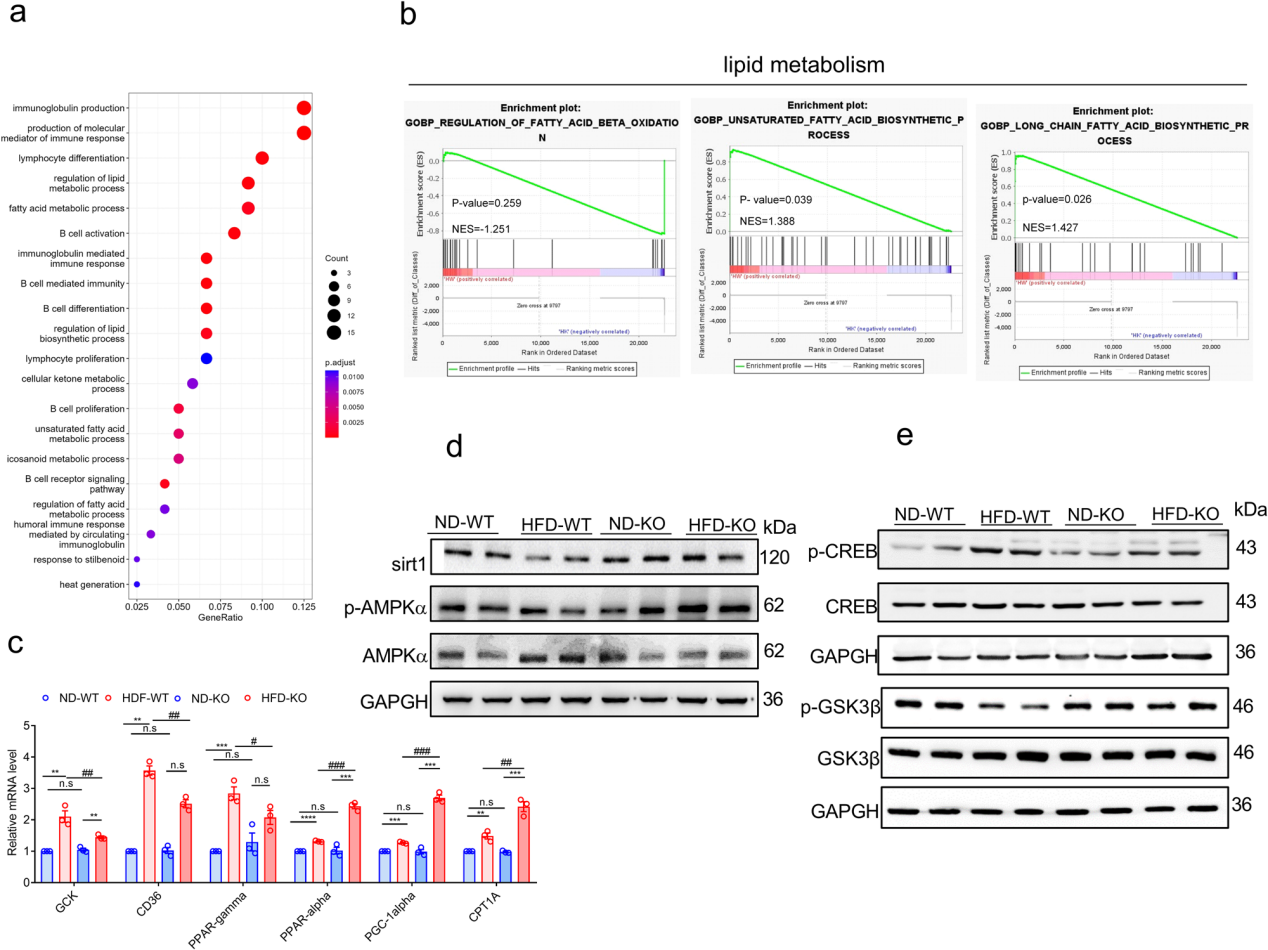
ADAR2 KO promotes activation of AMPK signaling in HFD-induced MAFLD of mice. **a** Gene ontology analysis showed the changed gene enrichment in the major pathway. **b** Gene set enrichment analysis showed the enrichment of signaling pathways associated with lipid metabolism in the livers of HFD-WT and HFD-KO mice after 12 weeks of the HFD feeding (*n* = 6 mice/group). **c** Relative mRNA expression levels of PPARα, PGC1α, and CPT1A in the liver were measured by qPCR (*n* = 6 samples/group, three independent experiments). **d** Relative protein expression levels of p-AMPK, AMPK, and sirt1 in the liver were determined by western blotting (*n* = 6/group, three independent experiments). **e** Relative protein expression levels of p-GSK3β, GSK3β, p-CREB, and CREB in the liver were determined by western blotting (*n* = 6/group, three independent experiments).

To systemically demonstrate the role of ADAR2 in NAFLD, we performed RNA-sequencing (RNA-seq) analysis in liver tissues from ADAR2 KO mice and WT mice after 12-week HFD feeding. Gene ontology (GO) functional enrichment analysis showed that metabolic biological pathways such as regulation of lipid metabolic process, fatty acid metabolic process, and regulation of fatty acid metabolic process were upregulated in livers of ADAR2 KO mice fed with HFD (HK) as compared with WT mice fed with HFD (HW) (Fig. 7a). Further, Gene-set enrichment analysis also showed that signaling pathways related to lipid metabolism such as regulation of fatty acid beta-oxidation upregulated, whereas the biosynthesis process such as unsaturated fatty acid and long chain fatty acid biosynthetic process downregulated in the HK group as compared with HW group. (Fig. 7b). We further explored the mechanism underlying the role of ADAR2 in lipid metabolism. ADAR2 KO significantly downregulated the levels of proteins involved in fatty acid synthesis (Fig. 4c). In contrast, ADAR2 KO increased the levels of mRNA involved in fatty acid β-oxidation, such as Peroxisome proliferator-activated receptor alpha (PPARα), peroxisome proliferator-activated receptor-gamma coactivator-1 alpha (PGC-1α), and carnitine palmitoyltransferase 1A (CPT1A) in the livers of HFD mice (Fig. 7c). As shown in Fig. 7d, protein expression of Sirt1 and the ratio of p-AMPK/AMPK were markedly increased in the liver of ADAR2 KO mice fed with HFD. These results indicate that ADAR2 KO regulates lipid metabolism by activating the AMPK/Sirt1 signaling pathway. The phosphorylation level of CREB was significantly reduced in the liver of ADAR2 KO mice fed with HFD compared with that of the WT mice fed with HFD (Fig. 7e). Furthermore, ADAR2 KO also increased the phosphorylation level of GSK3β (Fig. 7e). These findings demonstrated that ADAR2 KO partially reduced hepatic gluconeogenesis and increased glycogen contents by the AMPK/CREB/GSK3β signaling pathway. To provide additional evidence that ADAR2 KO inhibited hepatic lipogenesis via AMPK activation, ADAR2-deficient Huh7 cells were treated with palmitic acid in the presence or absence of compound C, an AMPK inhibitor. Following treatment, compound C attenuated the effect of ADAR2 KO on lipid accumulation. Results showed that lipid accumulation was decreased in ADAR2 KD Huh7 treated with PA compared with naïve Huh7 cells treated with PA, while treatments with PA and compound C enhanced lipid accumulation in ADAR2 KD Huh7 cells (Supplementary Fig. 6a, b). Moreover, compound C attenuated the effect of ADAR2 KO on the protein abundance of SREBP1, ACC1, FAS, and SCD1 (Supplementary Fig. 6c).

## Discussion

ADARs were originally identified as RNA-editing enzymes that modify cellular and viral RNA sequences by adenosine deamination^25^. Recent advances indicated that ADAR1 plays a vital role in hepatic immune homeostasis and adipogenesis^22^. Xiang et al. showed that overexpression of ADAR1 ameliorates HFD-induced NAFLD by inhibiting NLRP3 inflammasome and subsequent inflammation^22^. Yu et al. reported that ADAR1 overexpression could inhibit adipocyte differentiation, with a decrease in cell size and intracellular lipid droplets^26^. ADAR2 is another member of the adenosine deaminase family. However, little is known about its role in NAFLD. Heterozygous ADAR2^+/−^ mice were phenotypically normal, but ADAR2^−/−^ mice became prone to seizures after P12 and died between P0 and P20^27^. Early postnatal death of mice lacking ADAR2 was averted after introducing the second GluR-BR allele^24^. ADAR2^−/−^/GluR-B^R/R^ mice appeared normal at all ages. In this study, we employed ADAR2^−/−^/Glu-RB ^R/R^ mice that carry the edited (mutant) version of GluR to feed with HFD and examined the influence of ADAR2 in lipogenesis, especially in NAFLD. Our finding is that ADAR2 KO improved HFD-induced glucose/lipid-metabolic dysfunctions and improved hepatic glucose/lipid metabolism in obese mice. We also used Huh7 cells treated with PA to further clarify the functional ADAR2 in lipid accumulation. Knockdown of ADAR2 in Huh7 cells could inhibit lipid generation, with a reduction in intracellular lipid droplets and the expression of lipogenesis-related genes. Together, we exposed the crucial role of ADAR2 in hepatic lipogenesis, which indicates that ADAR2 could be the potential therapeutic target for NAFLD.

Differences in the diet composition, feeding duration, and age to start HFD feeding may differentially affect the functions of ADAR2. Wang et al. showed that HFD feeding, combined with Nω-nitro-l-arginine methyl ester, hydrochloride (L-NAME) decreased the ADAR2 expression in the livers of NAFLD mice and that exercise resulted in an obvious reversal in the ADAR2 level, which is synchronous with the process of lipid accumulation and dissipation in the liver observed in the histology results. This study also demonstrated that overexpression of ADAR2 in HepG2 cells treated with OA could inhibit lipid generation, with a decrease in intracellular lipid droplets and the expression of lipogenesis-related genes. In contrast with previous research, our study demonstrated that ADAR2 expression was increased in the livers of mice fed with 12-week HFD. ADAR2 KO could inhibit hepatic lipid accumulation in HFD-induced obese mice and PA-treated ADAR2 knockdown Huh7 cells. Thus, we speculate the different diet composition seems to affect the ADAR2 expression on NAFLD phenotypes. The seemingly contradictory effect may also be due to differences in ADAR2 expression that could affect RNA editing activities and the RNA target within the liver. Further studies of this remain to be investigated. In the present study, ADAR2 KO ameliorated the HFD-induced NAFLD phenotype in obese mice. As far as we know, this is the first research to investigate the hepatoprotective function of ADAR2 in HFD-induced NAFLD mice.

Sex differences exist in the susceptibility of NAFLD. However, the results of several epidemiological studies are still rather contradictory and the mechanisms involved are not yet clarified. The studies of Pinidiyapathirage et al.^28^ and Zhang et al.^29^ suggest a higher prevalence of NAFLD and NASH in men than in women. In contrast, the studies of Fernandes et al. conducted in Brazil^30^ and that of Haentjens et al. performed in Belgium^31^ show that women and girls, especially when being obese, are at higher risk of developing NAFLD than men. Furthermore, a study carried out in a mouse model in which mice were exposed chronically to 30% fructose solution to induce NAFLD suggests that female mice are more susceptible to the development of NAFLD^32^. In the present study, there is a marked weight gain in both male and female mice after HFD feeding, and ADAR2 KO reduced HFD-induced weight gain in both genders. Interestingly, ADAR2 KO reduced HFD-induced liver tissue weight gain and hepatic lipid accumulation in male mice but not in female mice (Supplementary Fig. 7). Indeed, glucose intolerance and insulin resistance were improved in male ADAR2 KO mice fed with HFD, while not observed in female ADAR2 KO mice fed with HFD. Taken together, these data suggest that female mice are more resistant to ADAR2 deficiency compared to male mice of the same strain under HFD feeding. However, the underlying mechanisms by which ADAR2 KO improved HFD-induced NAFLD in male mice but not in female mice, respectively, will have to be clarified in future studies.

AMP-activated protein kinase (AMPK) is a phylogenetically conserved serine/threonine protein kinase that has been proposed to act as an important energy sensor that regulates metabolic homeostasis. High-fat diet feeding reduces the expression and phosphorylation of AMPK in multiple tissues, including skeletal muscle, heart, liver, adipose tissue, aortic endothelium, and hypothalamus^33–35^. Activation of hepatic AMPK leads to increased glucose uptake and fatty acid oxidation and inhibition of fatty acid synthesis, cholesterol synthesis, and gluconeogenesis^36^. Hepatic AMPK activity is generally suppressed in both NAFL and NASH^37^. Our results showed that p-AMPK/AMPK protein expression was increased in the liver of ADAR2 KO mice fed with HFD compared with that of WT mice fed with HFD, indicating that ADAR2 KO improved hepatic impaired lipogenesis and gluconeogenesis via reactivation of AMPK. AMPK and Sirtuin 1 (Sirt1) are closely associated with lipid metabolism and activate each other in a finely tuned network^38^. Phosphorylation of AMPK changes the nicotinamide adenine dinucleotide (NAD^+^) abundance and the NAD^+^/NADH ratio to upregulate Sirt1^39^, which in turn plays favorable roles in regulating hepatic lipid metabolism, hepatic oxidative stress, and hepatic inflammation through deacetylating some transcriptional regulators against the development of fatty liver diseases^40^. Treatment*s* that enhance AMPK*/*Sirt1 expression to inhibit ACC activity and to increase lipolysis and β*-*oxidation could improve NAFLD in HFD-fed mice^41,42^. In this study, we showed for the first time that ADAR2 KO induced the Sirt1/AMPK pathway in vivo. Moreover, ADAR2 KO reduced the expression of lipogenic genes such as SREBP1c, ACC, FAS, and SCD1 at protein levels and increased the mRNA levels of β-oxidation genes, including PPARα, PGC1α, and CPT1A.

Glucose metabolism is a very complex process regulated by many mechanisms. In this study, we used a T2DM mouse model to investigate the effects of ADAR2 KO on glucose metabolism in vivo. ADAR2 KO significantly reduced fasting blood glucose levels, suggesting that ADAR2 KO had an obvious regulatory role in glucose metabolism. Most glucose utilization and production are derived from hepatic gluconeogenesis in vivo. Hepatic gluconeogenesis is mainly regulated by some key enzymes, including PEPCK, G6Pase, fructose-bisphosphatase 1 (FBP1), and pyruvate carboxylase (PCX). As the rate-limiting enzymes of hepatic gluconeogenesis, the expressions of PEPCK and G6Pase were all significantly decreased in the liver of ADAR2 KO mice fed with HFD, which suggested that ADAR2 KO influenced hepatic gluconeogenesis. Additionally, we also found that ADAR2 KO could promote glycogen synthesis. CREB activity has been proposed to exert a key role in modulating hepatic gluconeogenesis^43^. Interestingly, activation of AMPK could also induce an increase in the phosphorylation level of GSK3β, which leads to an increase in the glycogen synthesis level^44^. The relative expression levels of p-AMPK/AMPK, p-GSK3β/GSK3β, and p-CREB/CREB were all significantly changed in ADAR2 KO mice fed with HFD. Taken together, our results suggested that ADAR2 KO could induce the downregulation of PEPCK and G6Pase activities to inhibit hepatic gluconeogenesis via the AMPK/CREB signaling pathway and promote glycogen synthesis by activating the AMPK/GSK3β signaling pathway.

NAFLD and T2DM are common comorbidities^45^, and hepatic steatosis often develops before insulin resistance, indicating a causal role of hepatic lipid accumulation in the pathogenesis of hepatic insulin resistance^46^. We found that ADAR2 KO increased hepatic insulin action without affecting the insulin sensitivity of other tissues and was sufficient to improve systemic insulin resistance and glucose intolerance. Previous studies have demonstrated that hepatic glycogen synthesis decreases during hepatic insulin resistance due to decreased PI3K/AKT signaling^47^. Our results showed that p-PI3K/PI3K protein expression was increased in the liver of ADAR2 KO mice fed with HFD compared with that of WT mice fed with HFD, indicating ADAR2 KO mice fed with HFD had better insulin sensitivity (Supplementary Fig. 8).

In conclusion, our study elucidates that ADAR2 plays an essential role in lipid metabolism and is also critically involved in the pathogenesis of NAFLD. Collectively, we demonstrated that ADAR2 KO ameliorates hepatic steatosis, reduces liver tissue weight and body weight, and significantly decreases lipid accumulation in the livers of obese mice by regulating the AMPK-mediated signaling cascade.

## Methods

### Animals

ADAR2^−/−^/ GluR-B^R/R^ mice were kindly provided by Prof. Bertrand CM Tan and maintained on a B6129S genetic background^24^. The heterozygous animals were intercrossed, producing the wild‐type (ADAR^+/+^/ GluR-B^R/R^) and homozygous mutant (ADAR2^−/−^/ GluR-B^R/R^) mice employed in this study. Under the care of National Cheng Kung University Institutional Animal Care, mice were housed (4–5 per cage) in a temperature- (temperature: 25  ±  2 °C; humidity: around 60–80%) and light-control environment under a 12:12 h light–dark cycle (lights on at 6:00 AM). All experimental procedures of animal studies were approved by the Institutional Animal Care and Use Committees (IACUC) of National Cheng Kung University (IACUC approval number: 106069). We have complied with all relevant ethical regulations for animal use. Male or female mice at the age of 5 weeks were randomly divided into two groups as follows: normal diet (ND) and high-fat diet (HFD) feeding groups. The ND mice were fed with a standard diet (Cat. #: 5010, LabDiet, St. Louis, MO, USA), and the HFD mice were fed a diet containing 60% kcal fat (Cat. #: 58Y1, TestDiet, St. Louis, MO, USA). The mice were grouped during experimentation. The experimental timelines and the details of the number of mice utilized in each experiment are listed in Supplementary Fig. 1.

### Intraperitoneal glucose tolerance test

Glucose tolerance was assessed by intraperitoneal glucose tolerance test (IPGTT)^48,49^. After 16-h fasting, mice were intraperitoneally injected with glucose solution (2 g/kg body weight). Blood samples were collected at 0,15, 30, 60, 90, and 120 min after injections. Glucose concentration was determined by using OneTouch® Ultra test strips and OneTouch® UltraEasy blood glucose meter (LifeScan, Milpitas, CA, USA).

### Intraperitoneal insulin tolerance test

Insulin tolerance was assessed by intraperitoneal insulin tolerance test (IPITT)^48,49^. Mice were given insulin (0.75 U/kg body weight) by intraperitoneal injection after 4-h fasting. Blood samples were collected from the tails of mice at 0, 30, 60, 90, and 120 min after insulin injection. Glucose concentration was determined by using OneTouch® Ultra test strips and OneTouch® UltraEasy blood glucose meter (LifeScan).

### Intraperitoneal pyruvate tolerance test

Pyruvate tolerance was assessed by intraperitoneal pyruvate tolerance test (IPPTT)^50^. Mice were intraperitoneally injected with sodium pyruvate (2 g/kg body weight) after 12-h fasting. Blood samples were collected from the tails of mice at 0, 30, 60, 90, and 120 min after sodium pyruvate injection. Glucose levels were measured by using OneTouch® Ultra test strips and OneTouch® UltraEasy blood glucose meter (LifeScan).

### Measuring fasting plasma levels of glucose and insulin

After 12-h fasting, the mice were anesthetized, and blood samples were collected from the cardiac puncture with heparinized capillary tubes. Plasma was collected after centrifuging the blood at 3000 × *g* for 10 min. Plasma glucose concentrations were measured by a commercial glucose-oxidase kit (Cat. #: 11538, BioSystems, Barcelona, Spain). Plasma insulin concentrations were measured by a commercial mouse insulin ELISA kit (Cat. #: 10-1247-01, Mercodia, Uppsala, Sweden).

### Calculating homeostasis model assessment insulin resistance index

The insulin resistance (IR) index was calculated as follows using the formula: fasting glucose (mM) × fasting insulin (mU/l)/22.5^48,49^.

### Circulating lipid concentration measurements

The plasma specimens were directly applied to commercial quantification colorimetric kits to detect the selected plasma lipid levels. Commercial quantification colorimetric kits were employed to measure the circulating triglycerides (K622-100, BioVision, Milpitas, CA, USA), free fatty acid (K612-100, BioVision), HDL (K613-100, BioVision) and LDL/VLDL (K613-100, BioVision).

### Cell culture and cell treatment

Human hepatocellular carcinoma cell line Huh7 was kindly provided by Prof. Ming-Hong Tai^51,52^ and cultured in DMEM medium supplemented with 10% heat-inactivated fetal bovine serum (FBS) and 1% penicillin–streptomycin solution. The culture was maintained at 37 °C in a humidified 5% CO_2_ incubator. Huh7 cells were cultured in the presence or absence of 0.25 mM palmitic acid for 24 h and then used for the indicated assays. All the cell lines in this study were tested for mycoplasma. The cell lines with free contamination of mycoplasma were subjected to consequently experiments.

### Generation of ADAR2 knockdown (ADAR2 KD) Huh7 cells

ADAR2 knockdown cells were established by using shRNA lentiviral transduction, followed by puromycin selection. The pLKO-ADAR2-shRNA1 (5’CCCGTGATGATCTTGAACGAA3’), pLKO-ADAR2-shRNA2 (5’ CGGAGATCCTTGCTCAGATTT 3’) and pLKO-ADAR2-shRNA3 (5’ CCCAGGACTCAAGTATGACTT 3’) constructs were used for the lentivirus packaging.

### Oil Red O staining

Lipid droplet formation in cultured Huh7 cells was tested by using Oil Red O staining^53^. Huh7 cells were placed on coverslips overnight and then treated with 0.25 mM PA for 24 h. After PA treatment, cells were washed in PBS and then fixed in 4% paraformaldehyde for 30 min. The sample was briefly washed in isopropanol and stained with 60% Oil Red O solution (Sigma, Cat. #: 00625) for 1 h. The stained cells were washed in distilled water. The sample was then counterstained with hematoxylin (Leica, Cat #: 3801522) for 1 min and then observed under a bright field microscope.

### Antibodies, chemicals, immunoblotting

Antibodies against ACC (Cat. #: 3676), AKT (Cat. #: 9272), phospho-AKT (Cat. #: 4060), AMPKα (Cat. #: 5831), phospho-AMPKα (Cat. #: 2535), CREB (Cat. #: 9197), phospho-CREB (Cat. #: 9198), GSK3β (Cat. #: 5676), phospho-GSK3β (Cat. #: 9327), FAS (Cat. #: 3180), PEPCK (Cat. #: 12940), SCD1 (Cat. #: 2794), sirt1 (Cat. #: 8469) were from Cell Signaling Technology. Antibodies against G-6-P (Cat. #: ab83690) were from Abcam. Antibodies against GAPDH (Cat. #: MA5-15738) were from Thermo Fisher. Antibodies against SREBP-1(Cat. #: sc-13551) were from Santa Cruz. For western blotting, liver tissue specimens of mice were lysed with ice-cold commercial tissue protein extraction reagent (Cat. #: 78510, Thermo Fisher Scientific, Waltham, MA, USA) containing complete protease inhibitor cocktail tablets (Cat. #: 04693132001, Roche, Basel, Switzerland). Protein concentrations were measured by a Bio-Rad protein assay. An equal amount of protein lysates was separated by SDS–PAGE and then transferred to a nitrocellulose membrane (Cat. #: S80209, Pall Life Science, Port Washington, NY, USA). Membranes were blocked in blocking buffer and then incubated with primary antibodies, washed, and incubated with the corresponding horseradish peroxidase-conjugated secondary antibodies (Cat. #: 115-035-003 and 111-035-003, Jackson ImmunoResearch, West Grove, PA, USA), and visualized with western blotting luminol reagent (Cat. #: sc-2048, Santa Cruz Biotechnology, Inc.). Bands in the immunoblots were quantified by using ImageQuant LAS 4000 (GE Healthcare, Chicago, IL, USA).

### Analysis of tissue lipid droplets

Liver, white adipose tissue (WAT), and brown adipose tissue (BAT) isolated from mice (*n* = 5) were fixed at 4% paraformaldehyde, embedded in paraffin, and cut into 5 μm sections. Specimens were deparaffinized, hydrated, and stained as a usual method with standard hematoxylin and eosin staining (H&E). The analysis was done with the measurement tool within the ImageJ software. For each mouse, 500–1000 lipid droplets were counted. Five random fields per tissue were evaluated.

### In vivo insulin sensitivity in mice

Mice were fasted for 24 h and then given five units of insulin (Novo Nordisk, Bagsvaerd, Denmark) in the inferior vena cava. Tissues (liver (WAT) and muscle (BAT)) were harvested from the injected animals in each group after 5 min and snap frozen. Whole-cell extracts were prepared from each tissue to measure insulin-dependent phosphorylation of Akt by western blot analysis.

### Isolation of mouse primary hepatocytes

Primary hepatocytes were isolated from the livers of 6- to 8-week-old male mice by a two-step collagenase perfusion process. Briefly, after anesthetization, mice were perfused through the portal vein with Ca^2+^ and Mg^2+^ free Hank’s balanced salt solution (HBSS) with 0.5 mM EGTA and then perfused with HBSS containing collagenase type I. After digestion, the livers were excised, minced, and filtered through a 100-µm cell strainer. Hepatocytes were separated by centrifugation at 100 × *g* for 5 min two times. The obtained hepatocytes were resuspended in Dulbecco’s modified Eagle’s medium (DMEM) supplemented with 10% fetal bovine serum and 1% penicillin/streptomycin and seeded on collagen-coated dishes (BD Falcon, 35 mm).

### Glucose production assay

Cells were washed twice with PBS to remove glucose and incubated in 500 µl glucose-free culture medium with and without 2 mM pyruvate, and 20 mM lactate for 4 h. Glucose concentrations were measured with glucose oxidase assay kit (BioSystems, Cat: #11538) and normalized with protein concentrations. Protein content was determined using a Bio-Rad protein assay.

### Glucose uptake assay

Glucose uptake assay was performed by using a glucose uptake cell-based assay kit (Cayman, Cat: #600470) according to the manufacturer’s instructions. After cells were cultured with 0.25 mM PA in glucose-free culture medium for 24 h, cells were treated with or without 200 nmol/l insulin for 30 min. Glucose uptake was performed by incubation with a glucose-free culture medium containing 2-NBDG (200 μg/ml) for 30 min and terminated by treatment with cell-based assay buffer before detecting fluorescein (excitation/emission = 485/535 nm).

### Liver glycogen content

Livers isolated from mice (*n* = 5) were fixed at 4% paraformaldehyde, embedded in paraffin. Livers were sectioned at 5 μm and afterward deparaffinized and rehydrated. The Periodic Acid–Schiff (PAS) stain was performed with periodic acid and Schiff reagent (Sigma-Alddrich, Cat: #101646).

### RNA isolation and transcriptome sequencing

TRIzol reagent (Invitrogen) was used for RNA extraction, and cDNA libraries were prepared based on the MGIEasy RNA Directional Library Prep Kit Guide (MGI Tech, Cat. 1000006385). Equal concentrations of each library were sequenced using a DNBSEQ-G400RS (MGI Tech) platform to create pair-end 100-bp reads. Quality assessment and trimming of the generated sequences were done by the RNA-seq alignment tool from MGI Tech, followed by alignment to the mouse reference genome (mm10). The expression levels of genes in each sample and the corresponding fold changes were estimated by Partek Genomics Suite with GENCODE annotation. Relative expression of each gene is represented by counts per million (CPM). Partek Genomics Suite and statistical package were used for the statistical analysis, hierarchical clustering differential expression analysis.

### Gene ontology (GO) analysis

To further analyze the differences in gene functions and pathways between WT mice fed HFD and ADAR2 KO mice fed with HFD, Gene Ontology (GO) was performed on the upregulated genes (Log fold change ≥ 1) in DEGs using the *enrichGO* function in the clusterProfiler package in R (v4.8.1). The significant enrichment terms were obtained by Benjamini-Hochberg (BH) with an adjusted *p*-value < 0.05.

### Gene set enrichment analysis

We used GSEA Desktop Application v4.3.2 to perform gene set enrichment analysis (GSEA) for our datasets. We chose the mouse collections MH, M2, and M5 gene sets from the Molecular Signatures Database (MSigDB v2023.1.Mm), and the phenotype label was set as HW (WT mice) vs. HK (KO mice). The minimum and maximum criteria for gene sets from the collection were between 15 and 500 genes, and the upregulated pathways were defined by an enrichment score (ES) > 0 and the downregulated pathways were defined by ES < 0, respectively.

### Real-time quantitative PCR analysis

Total RNAs were extracted using TRIzol Reagent (Cat. #: 15596018, Ambion, Life Technologies Corporation). The reverse transcription reaction was performed on 2 μg of RNA using SuperScript III First-standard Synthesis System (Cat. #: 18080-051, Invitrogen) with random hexamer primers according to the manufacturer’s directions. SYBR Green (Cat. #: 4385612, Thermo Fisher Scientific) was used to quantify the PCR amplification products. The expression levels of the target mRNA were measured using a real-time PCR system (Applied Biosystems, Thermo Fisher Scientific). The target mRNA levels were normalized to the levels of the housekeeping gene β-actin, which was used as the endogenous control. The sequences of the primers are listed in Supplementary Table 1.

### Statistical analysis

All data were plotted and reported as mean ± standard error of the mean (SEM). Significance was set at *p* < 0.05. Student’s *t* test was adopted to analyze the data sets with a single factor (HFD effect). The body weight and energy intake of mice were analyzed using repeated measured two-way ANOVA. Ordinary two-way ANOVAs were used to analyze the results with two factors (HFD and ADAR2 KO). Sidak’s post-hoc test was used to perform multiple comparison analyses after the two-way ANOVAs. Statistical analyses were performed with GraphPad Prism 8.0 (GraphPad Software, Inc., San Diego, CA).

### Reporting summary

Further information on research design is available in the Nature Portfolio Reporting Summary linked to this article.

### Supplementary information

Supplementary Information

Description of Additional Supplementary Files

Supplementary Data 1

Reporting Summary

## Data Availability

The gene expression profile reported in this paper has been deposited in the Gene Expression Omnibus (GEO) database (GSE261185). The Source data are provided in Supplementary Data 1. Images of uncropped blots are provided in Supplementary Fig. 9. The data underlying this article will be shared on reasonable request to the corresponding author.
