# Supplementary material for: Deficiency of ADAR2 ameliorates metabolic-associated fatty liver disease via AMPK signaling pathways in obese mice
Source: Commun Biol. 2024 May 17;7:594. doi: 10.1038/s42003-024-06215-4 (PMC11101631; doi:10.1038/s42003-024-06215-4)
Supplement: Supplementary file 1 — Supplementary Information [file 42003_2024_6215_MOESM1_ESM.pdf]

# Deficiency of ADAR2 ameliorates metabolic-associated fatty liver disease via AMPK signaling pathways in obese mice

†Mei-Lang Kung[1], †Siao Muk Cheng[2], Yun-Han Wang[3], Kai-Pi Cheng[4], Yu-Lin Li[3], Yi-Tsen Hsiao[3], Bertrand Chin-Ming Tan[5,6,7,8], Yun-Wen Chen[3*]

[1]Department of Medical Education and Research, Kaohsiung Veterans General Hospital, Kaohsiung, Taiwan.
[2]National Institute of Cancer Research, National Health Research Institutes (NHRI), Tainan, Taiwan
[3]Department of Pharmacology, College of Medicine, National Cheng Kung University, Tainan, Taiwan
[4]Department of Internal Medicine, National Cheng Kung University Hospital, College of Medicine, National Cheng Kung University, Tainan, Taiwan
[5]Graduate Institute of Biomedical Sciences, College of Medicine, Chang Gung University, Taoyuan, Taiwan.
[6]Department of Biomedical Sciences, College of Medicine, Chang Gung University, Taoyuan, Taiwan.
[7]Department of Neurosurgery, Linkou Medical Center, Chang Gung Memorial Hospital, Linkou, Taiwan.
[8]Research Center for Emerging Viral Infections, Chang Gung University, Taoyuan, Taiwan.

† These authors contributed equally

*Address correspondence to:
Yun-Wen Chen, PhD,
Department of Pharmacology, College of Medicine, National Cheng Kung University, Tainan 701, Taiwan
Tel: 886-6-2353535 ext 5503; Fax: 886-6-2766185
E-mail: yunwen _chen@mail.ncku.edu.tw

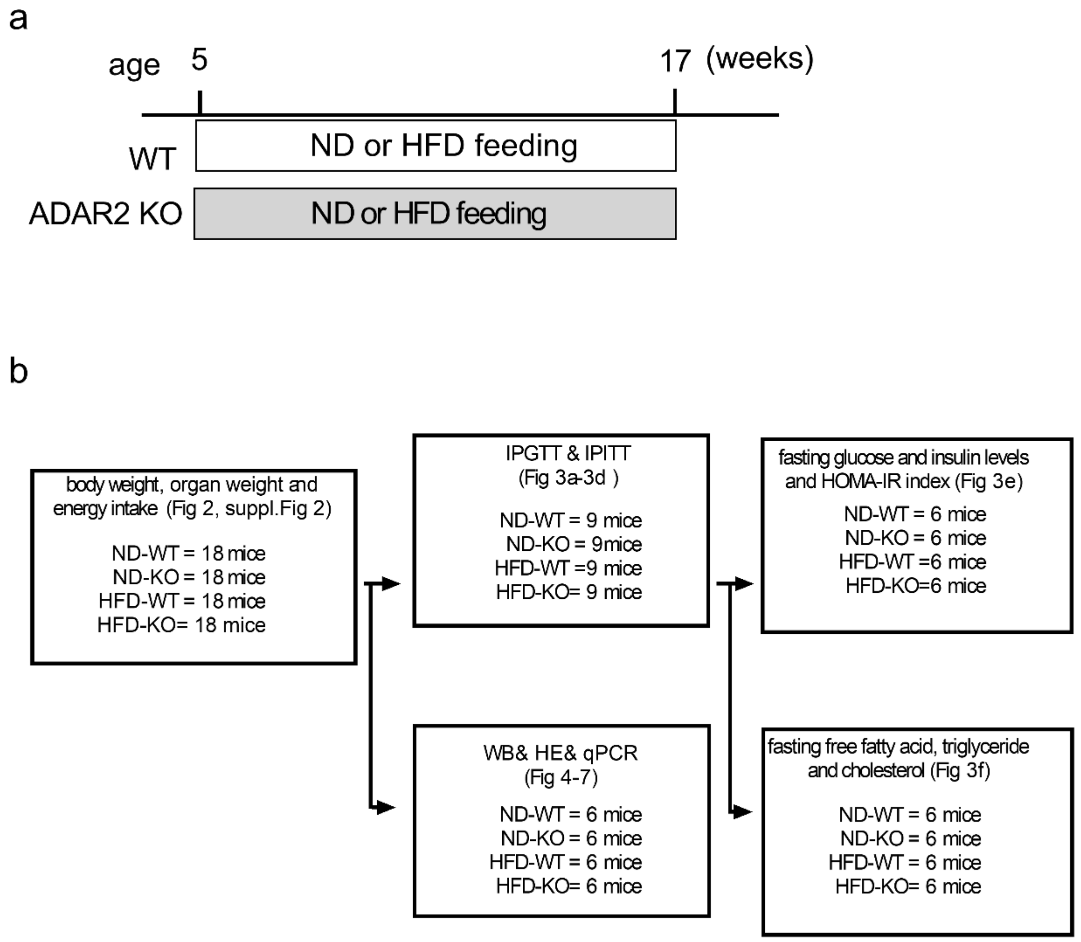


**Supplementary Figure S1. Details of number of animals used in each experiment.** (a) Experimental timeline. Mice are fed with standard chow or high-fat diet (HFD) at 5-week of age for 12 weeks. (b) Experimental procedures and number of animals used in each experiment. Body weight, food intake, and water intake were measured once every week. Energy intake is calculated based on food consumption per week (kCal/week). Intraperitoneal glucose tolerance test, and intraperitoneal insulin tolerance test were performed at 17-week of age. Blood samples for fasting glucose level, fasting insulin level, and fasting free fatty acid, fasting triglyceride, and fasting cholesterol were collected at 17-week of age.

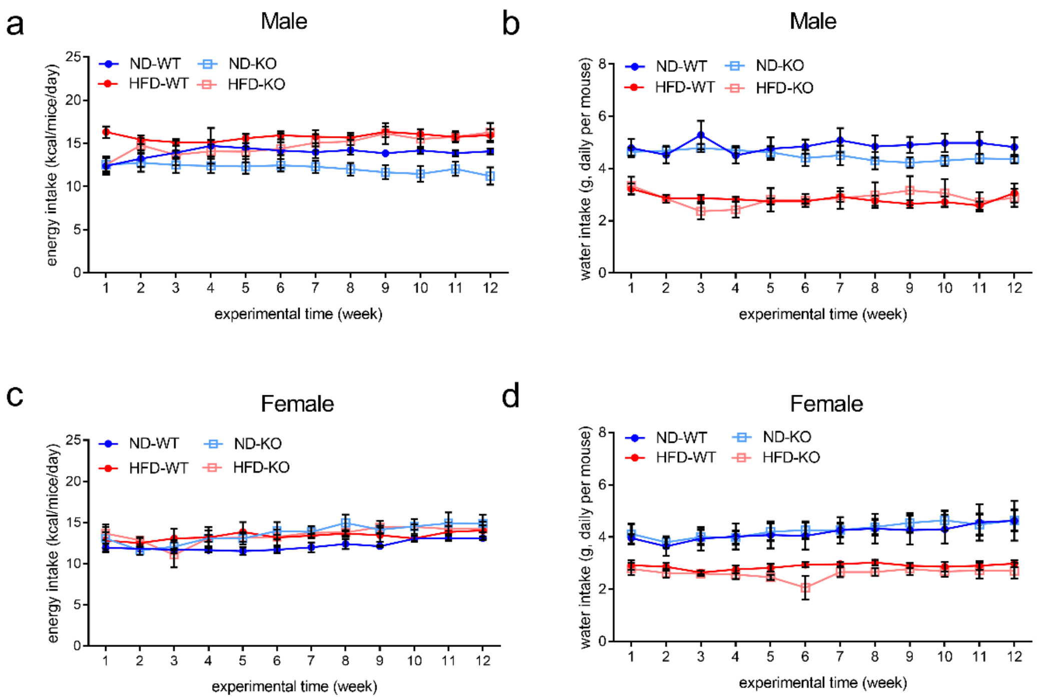


**Supplementary Figure S2. Effects of ADAR2 KO on energy intake and water intake.** Physiological parameters in both male and female mice from the age of 5 weeks to 17 weeks. n=18 mice, 5 cages (a,c) energy intake calculated based on food consumption per week (kCal/week); (b,d) water intake calculated based on water consumption per week

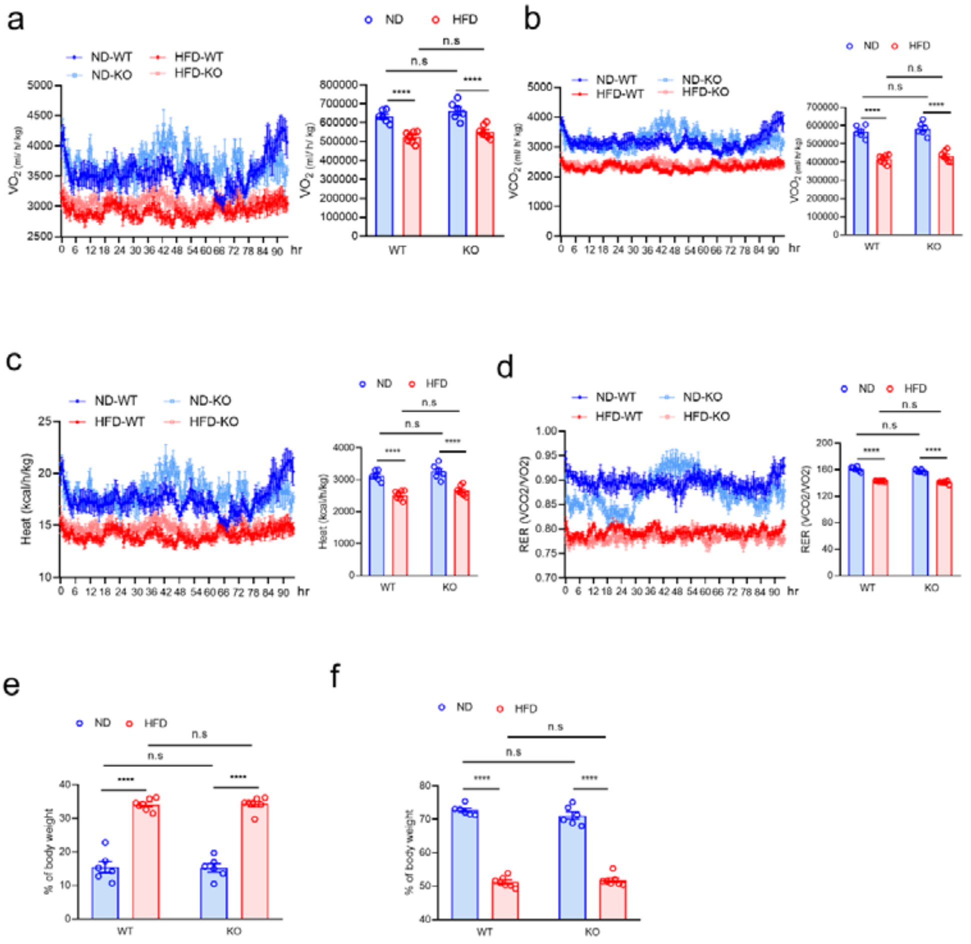


**Supplementary Figure S3. There was no significant difference on oxygen consumption, carbon dioxide generation, respiratory exchange ratio, energy expenditure/heat generation, lean mass, and fat mass of ADAR2-KO male mice fed with HFD when compared with WT male mice fed with HFD. (a)** Oxygen consumption, (b) carbon dioxide generation, (c) energy expenditure, (d) respiratory exchange ratio, (e) fat mass, and (f) lean mass of WT littermates and ADAR2 KO mice fed with either ND or HFD are shown. All data are expressed as mean ± S.E.M. Tukey's multiple comparison test after the two-way ANOVA was conducted for (a)- (f). *ND-WT group versus HFD-WT group or ND-KO group versus HFD-KO group; ****p < 0.0001; n.s, not significant. n=6-7 mice

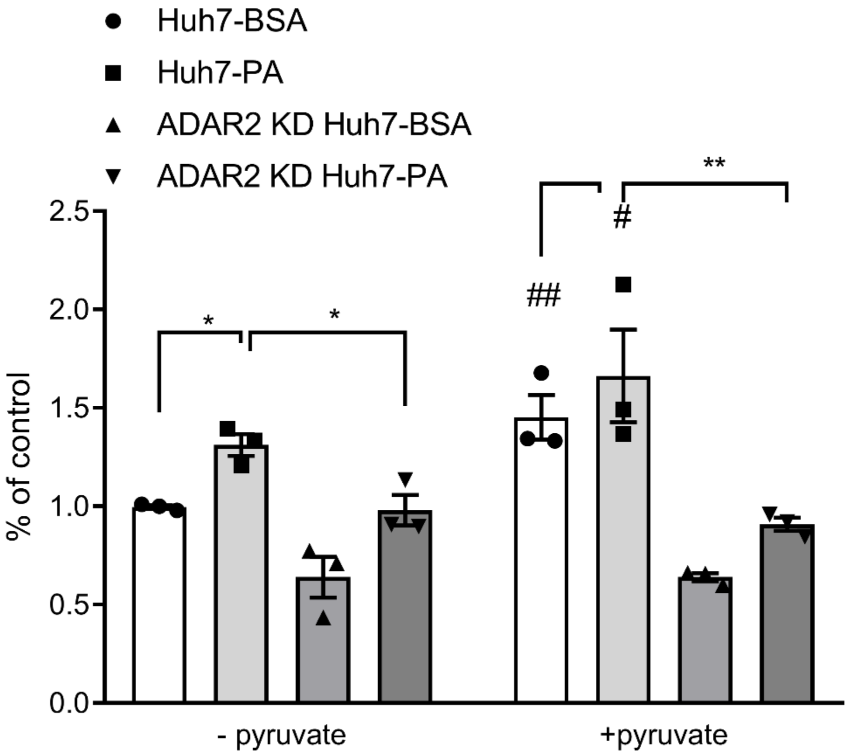


**Supplementary Figure S4. Pyruvate-induced glucose production in ADAR2-deficient Huh7 cells.** ADAR2-deficient Huh7 cells were treated with 0.25 mM PA for 24 hr. After PA treatment, cells were stimulated with or without pyruvate, and glucose production levels were measured by a glucose oxidase assay kit. Results are derived from three independent experiments performed in triplicate. *$p < 0.05$, ****$p < 0.001$, #$p < 0.05$, ##$p < 0.01$, ###$p < 0.001$,vs. without pyruvate group,

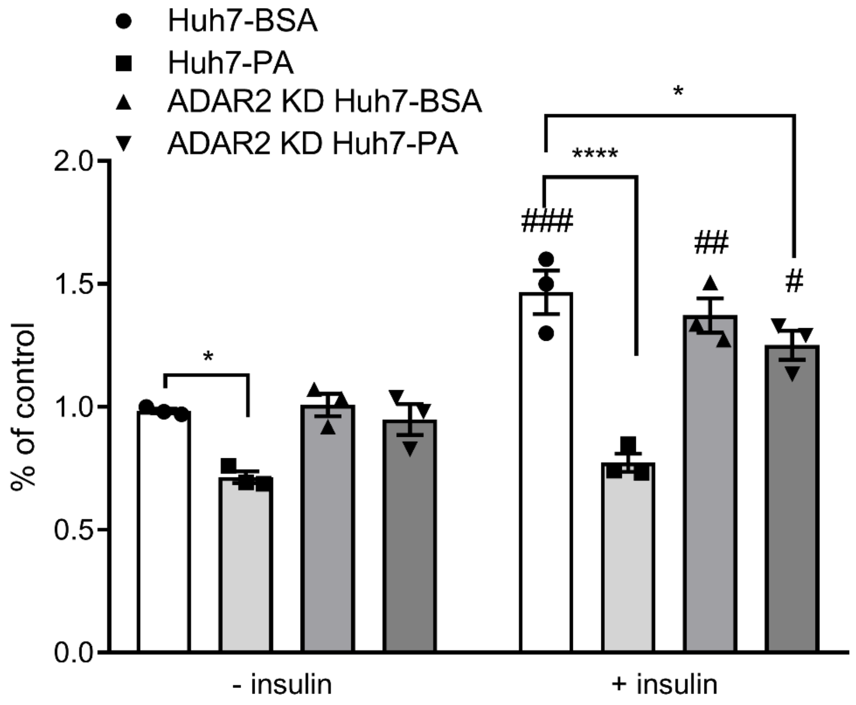


**Supplementary Figure S5. Insulin- stimulates glucose uptake in ADAR2-deficient Huh7 cells.** ADAR2-deficient Huh7 cells were treated with 0.25 mM PA for 24 hr. After PA treatment, cells were stimulated with or without insulin, and glucose uptake levels were measured by a glucose uptake cell-based assay kit. Results are derived from three independent experiments performed in triplicate. *p < 0.05, ****p < 0.001, #p < 0.05, ##p < 0.01, ###p < 0.001,vs. without insulin group,

a

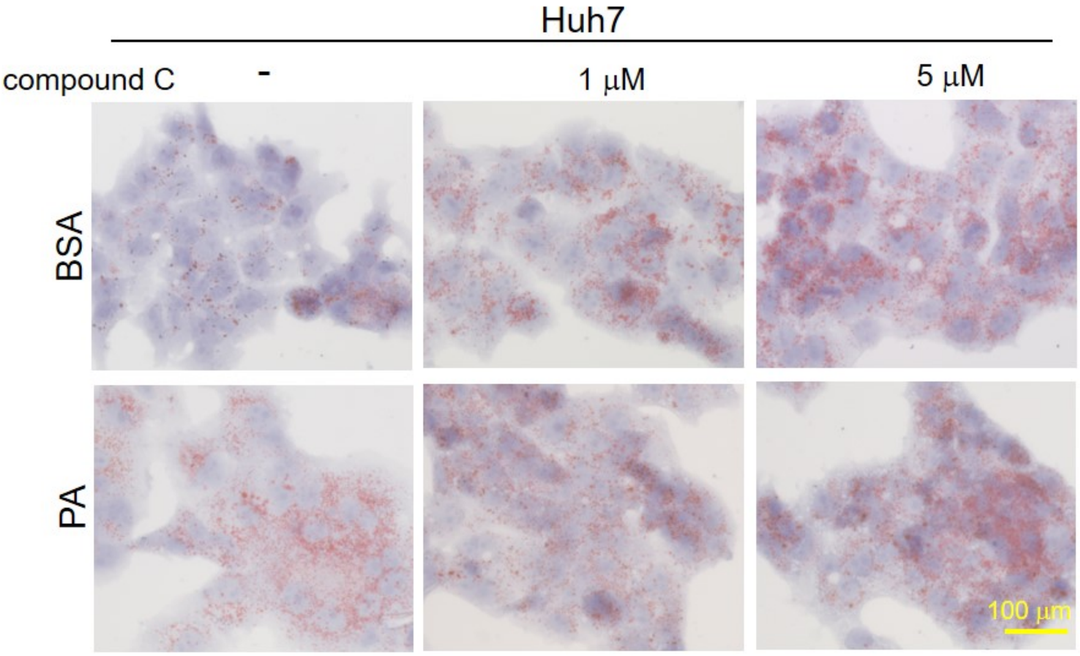

Huh7
compound C
-
1 μM
5 μM
BSA
PA
100 μm


b

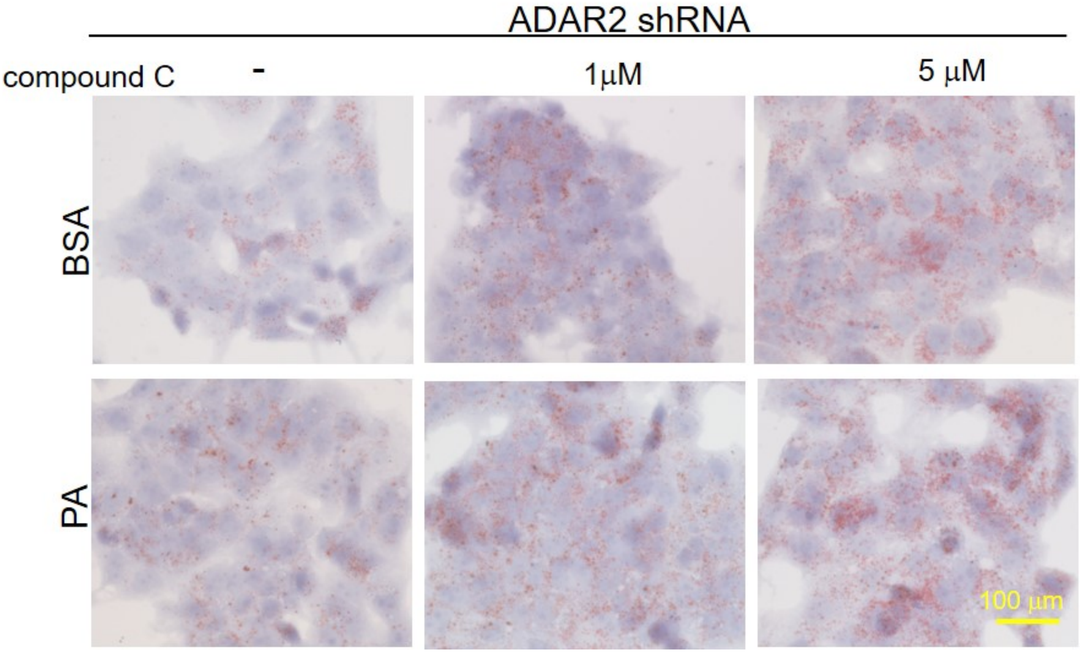

ADAR2 shRNA
compound C
-
1μM
5 μM
BSA
PA
100 μm

c

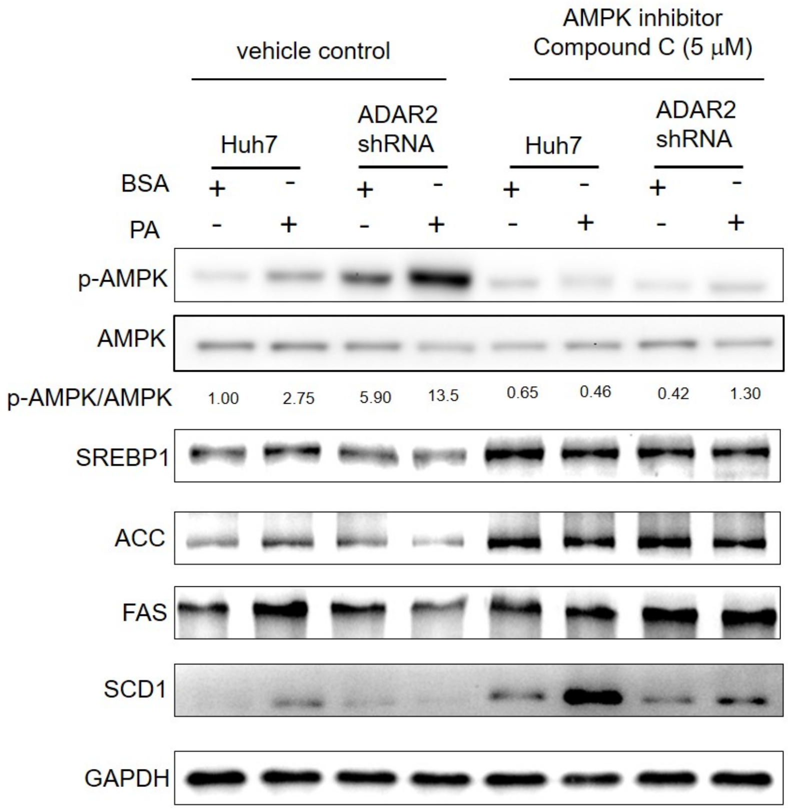


**Supplementary Figure S6.** Inhibition of AMPK by compound C increases ADAR2-mediated reduced hepatic lipogenesis. (a) Representative Oil red O image of lipid drop accumulation in Huh7 cells stimulated with palmitic acid (PA) (0.25 mM) with or without compound C for 24 hours. (n=3, three independent experiments). (b) Representative Oil red O image of lipid drop accumulation in ADAR2-deficient Huh7 cells stimulated with palmitic acid (PA) (0.25 mM) with or without compound C for 24 hours. (n=3, three independent experiments). Scale bars=100 µm. (c) Western blot of de novo lipogenesis-related protein in Huh7 cells or ADAR2-deficient Huh7 cells stimulated with palmitic acid (PA) (0.25 mM) with or without for 24 hours (n =3, three independent experiments).

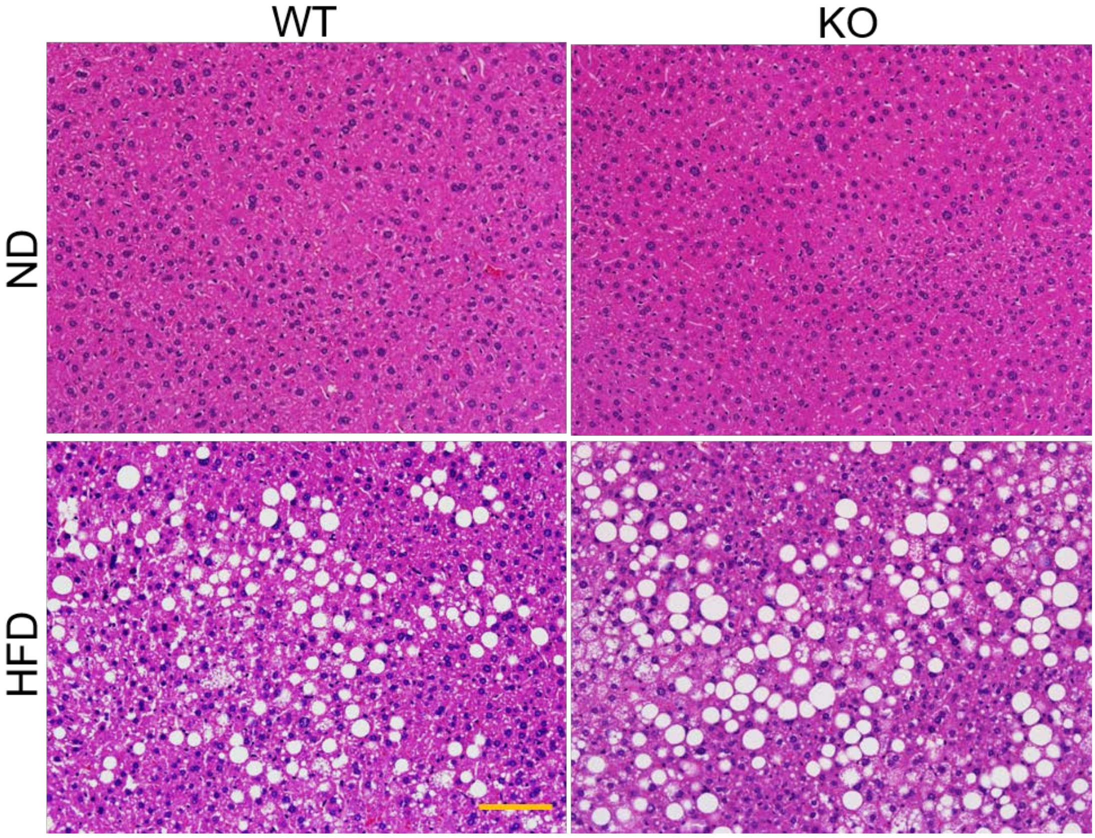


**Supplementary Figure S7. Effects of ADAR2 KO on lipid accumulation in the liver of female mice.** Representative H&E staining of the liver from WT and ADAR2 KO female mice fed with ND or HFD is shown (n = 8 mice/group). Scale bars=100 μm.

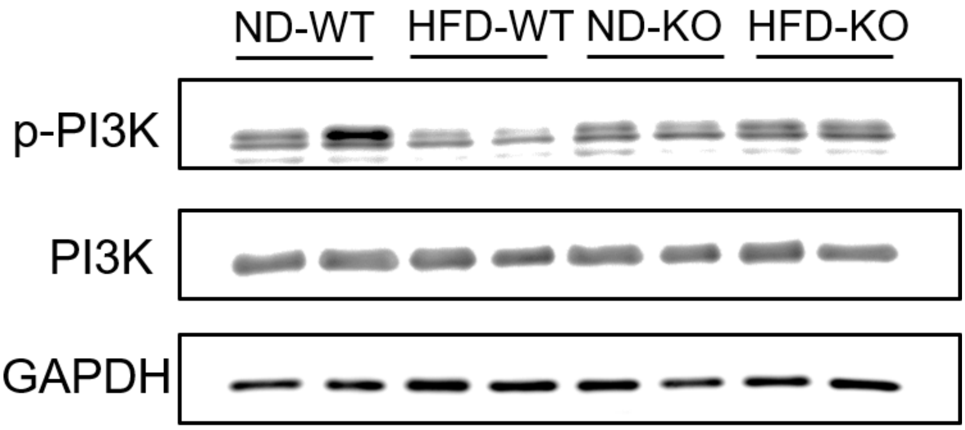


**Supplementary Figure S8. ADAR2 KO mice fed with HFD showed increased protein expression of p-PI3K/PI3K in the liver compared with that of WT mice with HFD.** Relative protein expression levels of p-PI3K and PI3K in the livers were determined by western blotting. n = 6 per group.

Supplementary Figure S9. Images of uncropped blots

Figure 1a

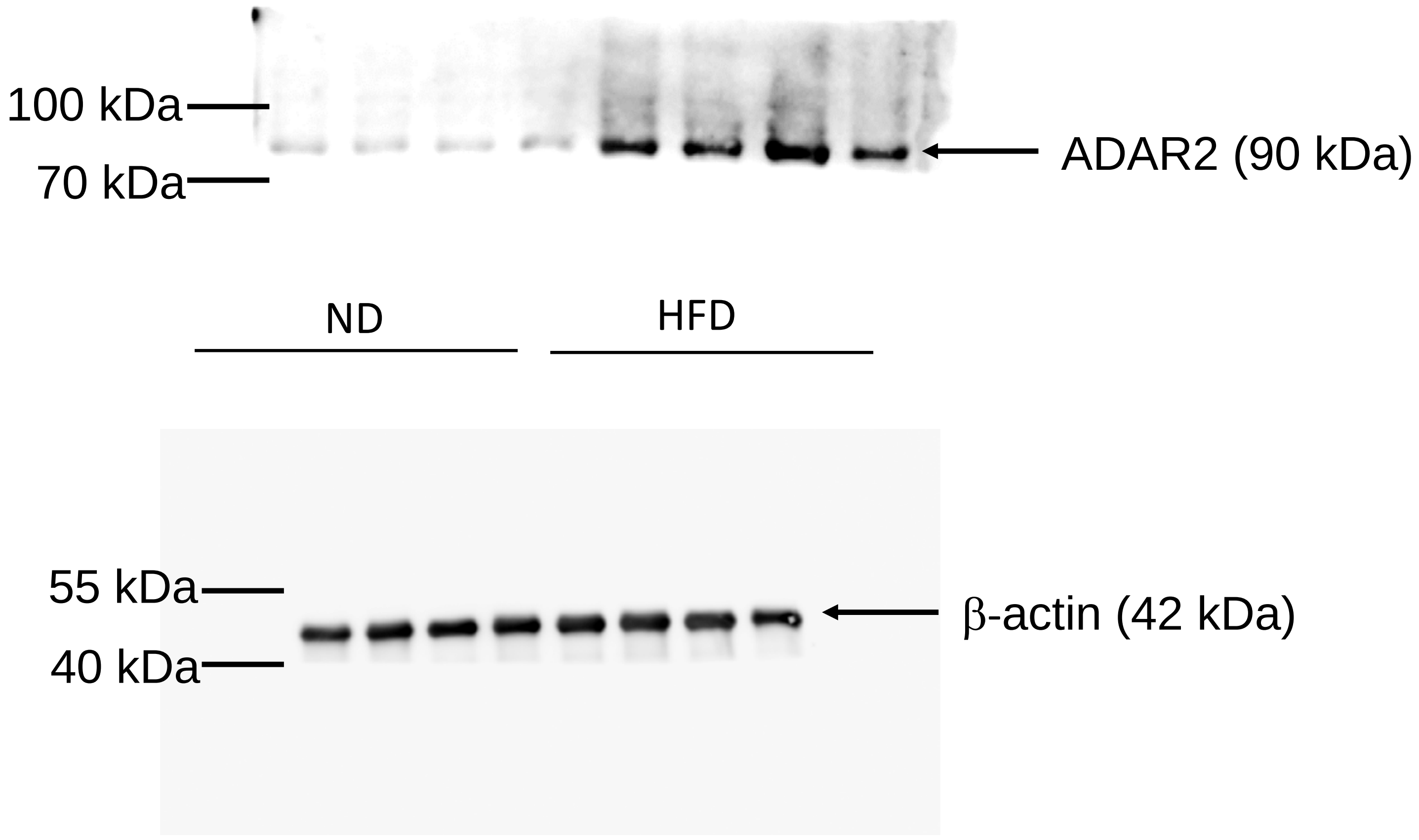

Figure 1b

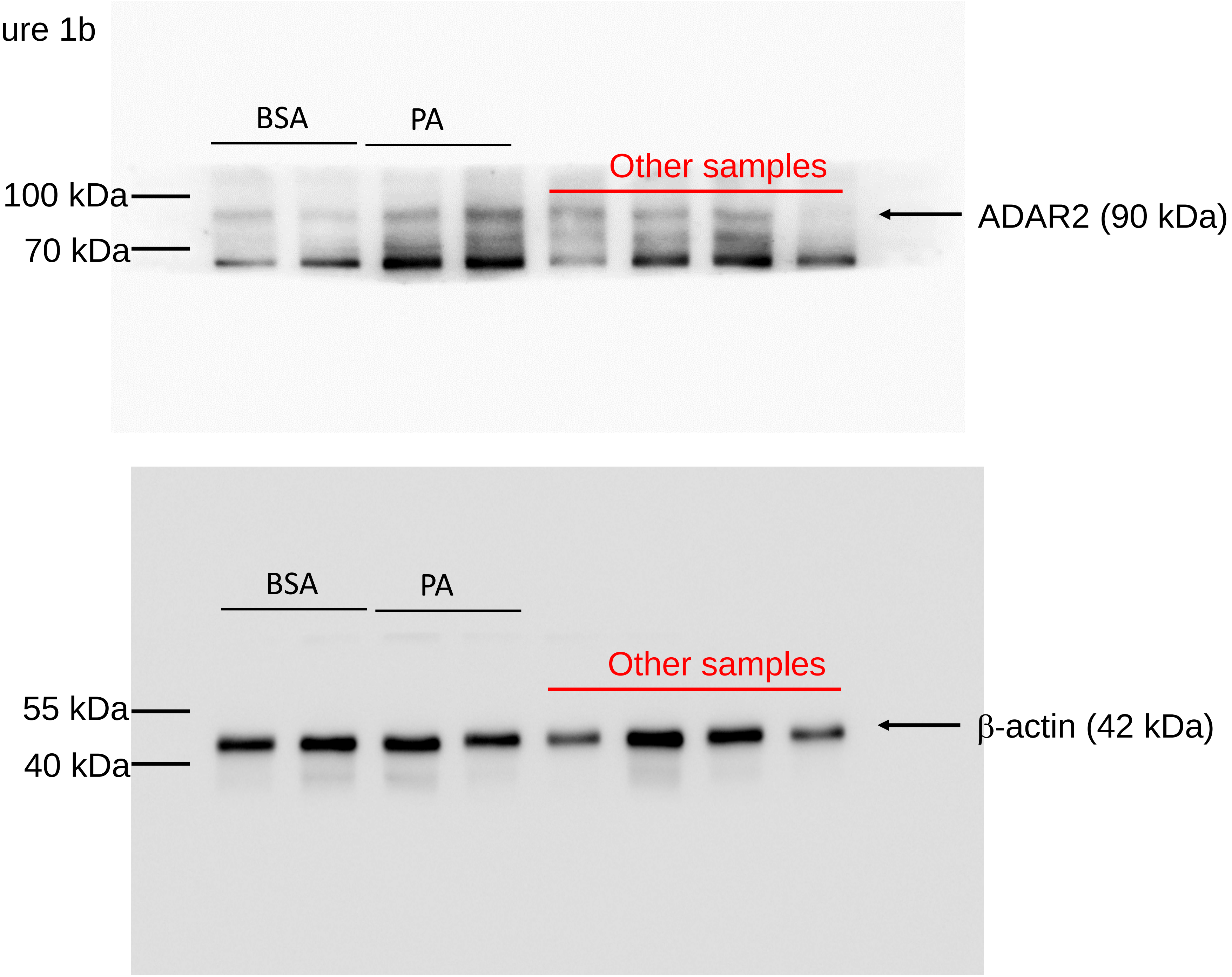
BSA
PA
Other samples
100 kDa
70 kDa
ADAR2 (90 kDa)
BSA
PA
Other samples
55 kDa
40 kDa
β-actin (42 kDa)

Figure 1c

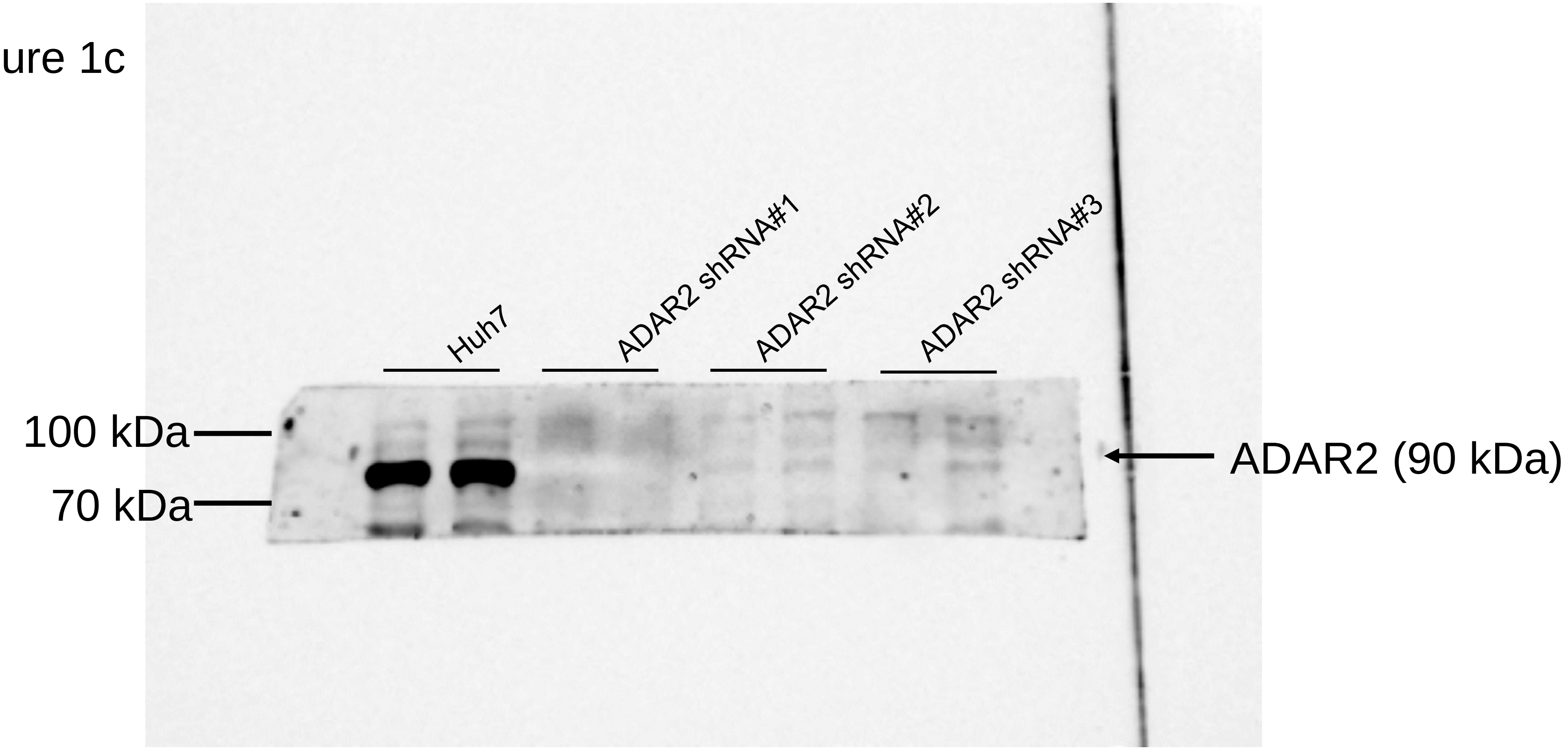
Huh7
ADAR2 shRNA#1
ADAR2 shRNA#2
ADAR2 shRNA#3
100 kDa
70 kDa
ADAR2 (90 kDa)

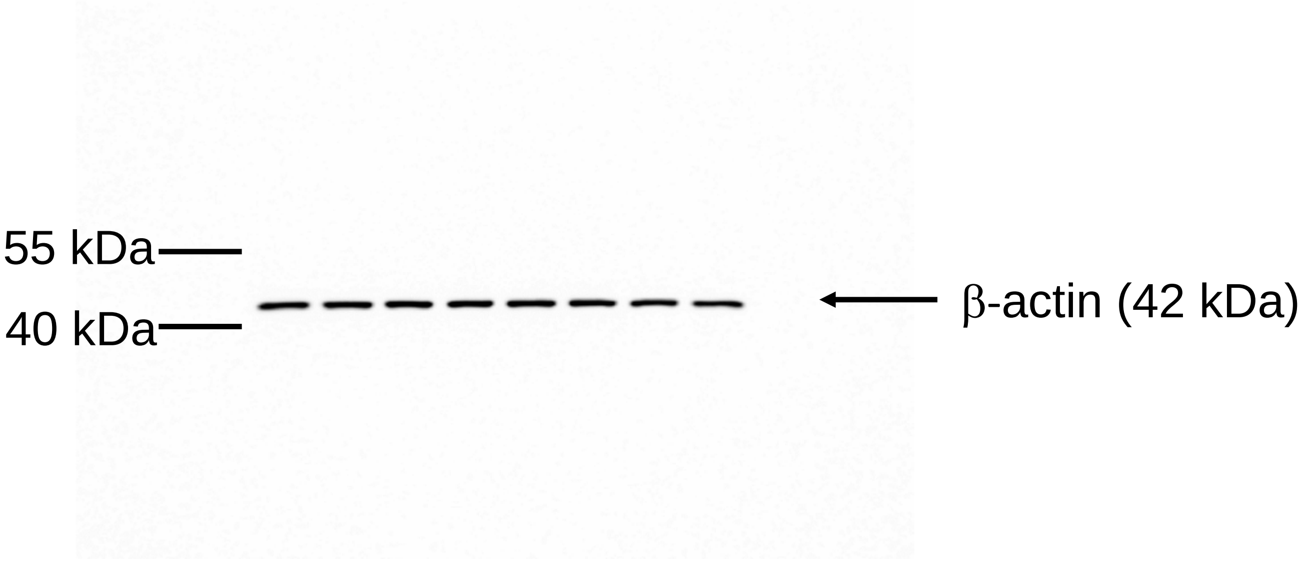
55 kDa
40 kDa
β-actin (42 kDa)

Figure 1e

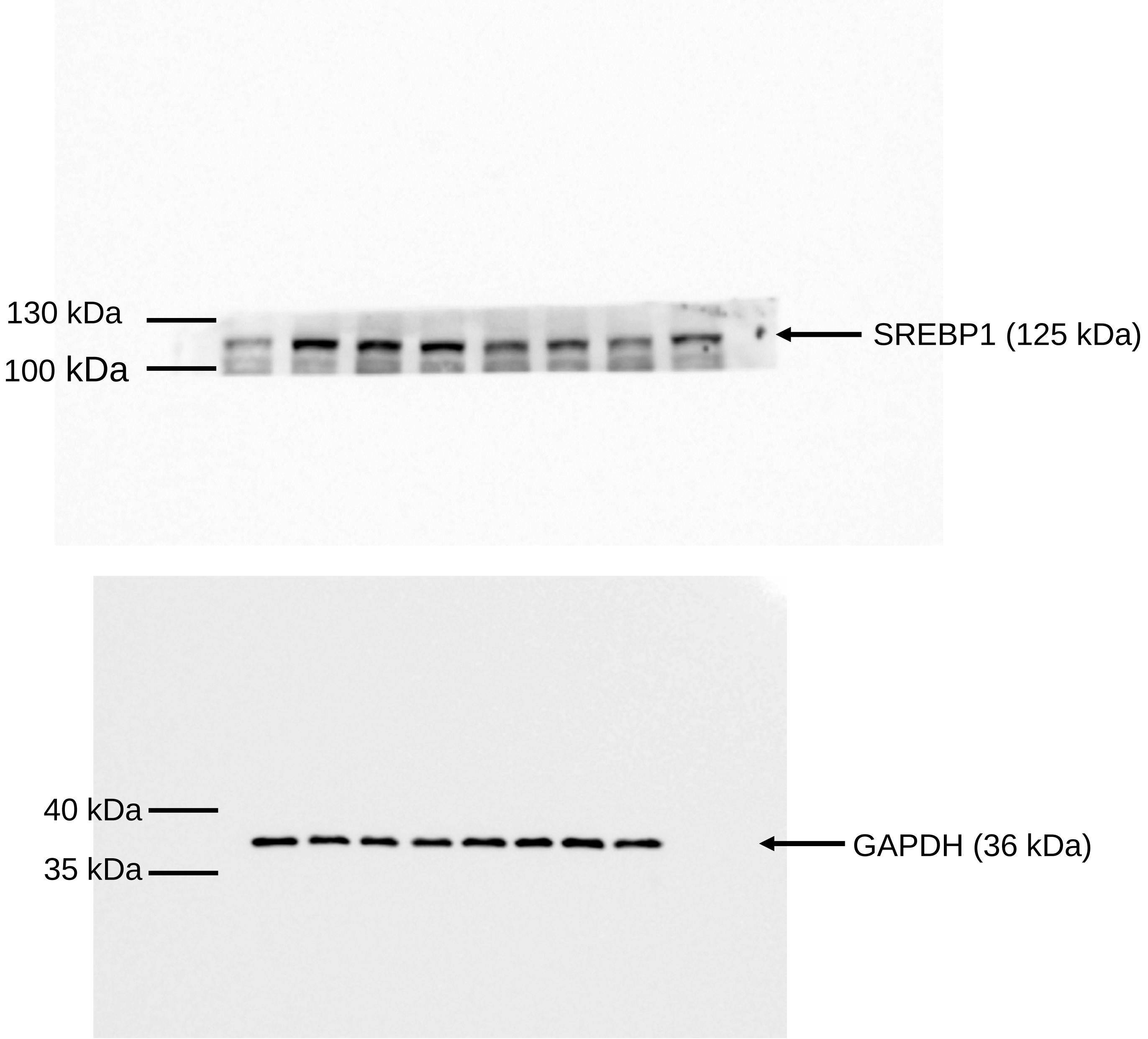

Figure 1e

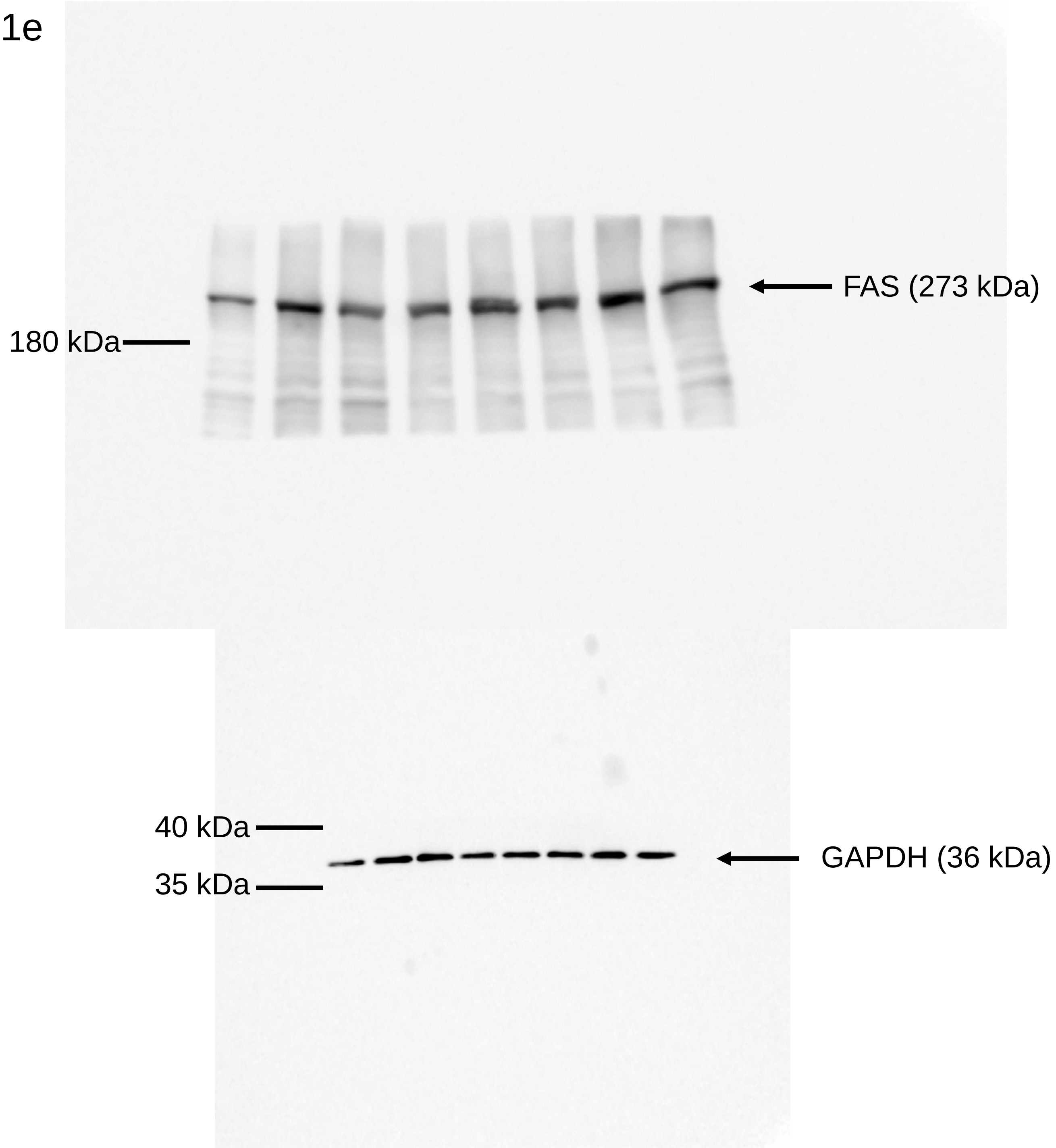

Figure 1e

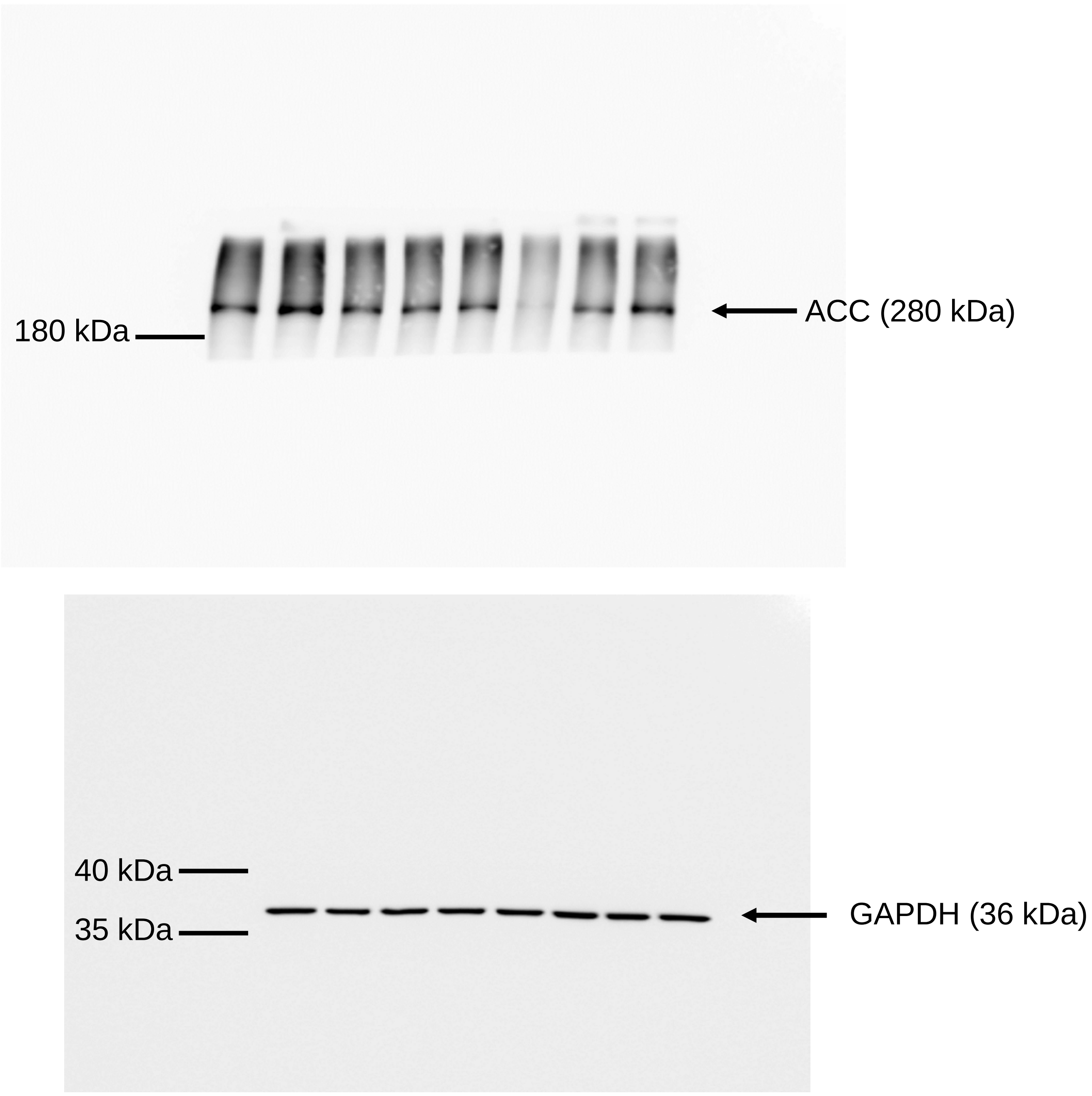

Figure 1e

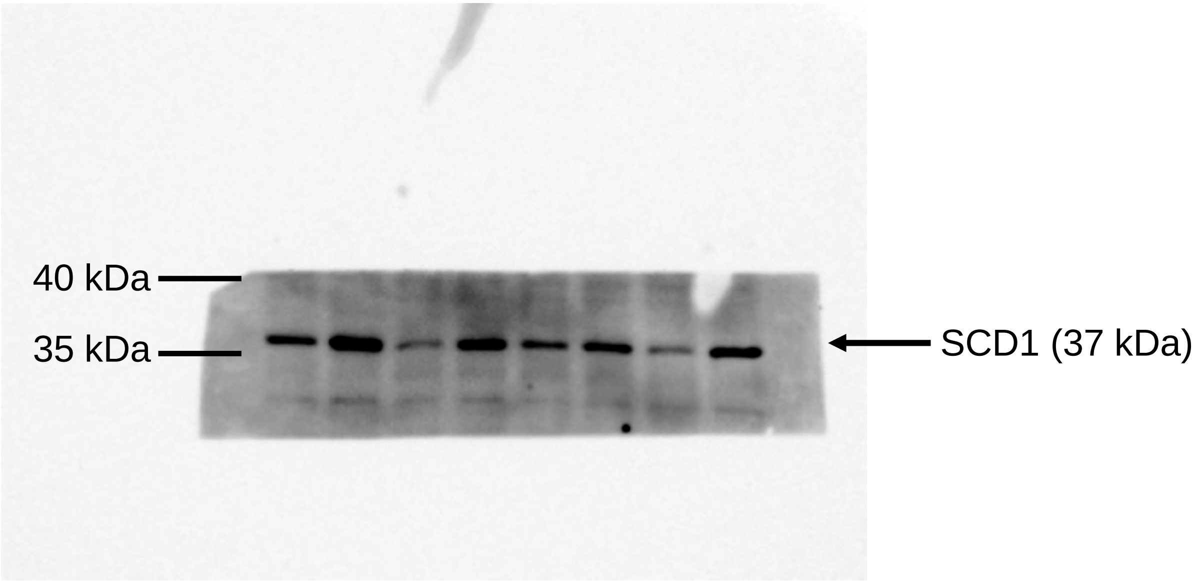


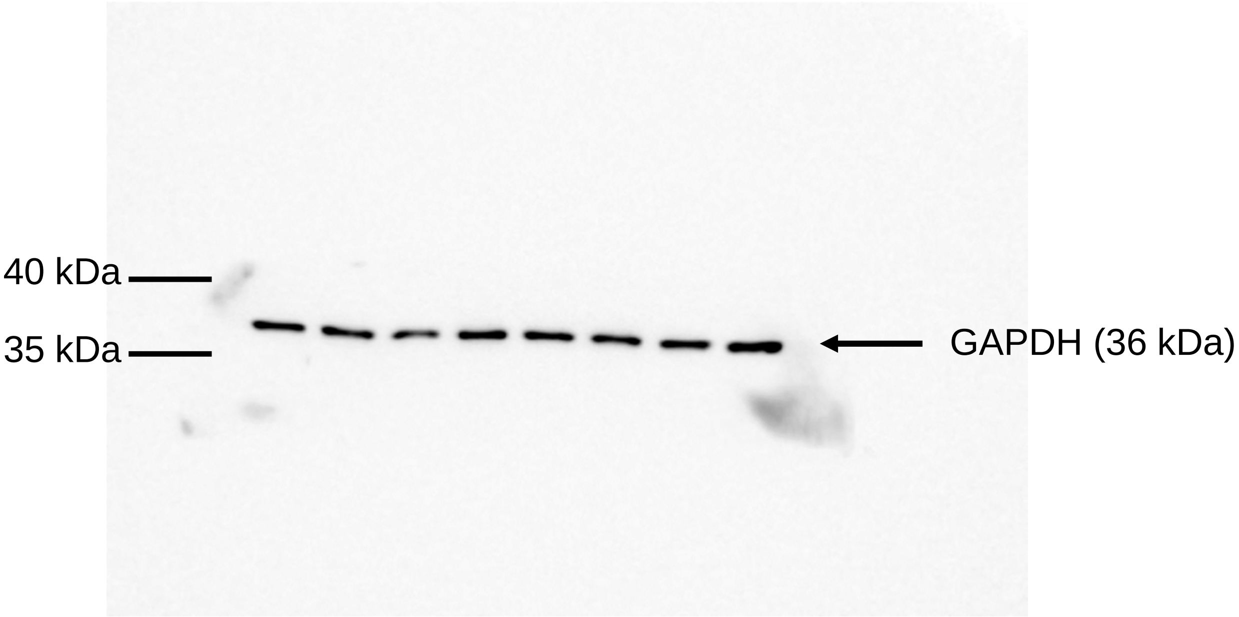

Figure 4c

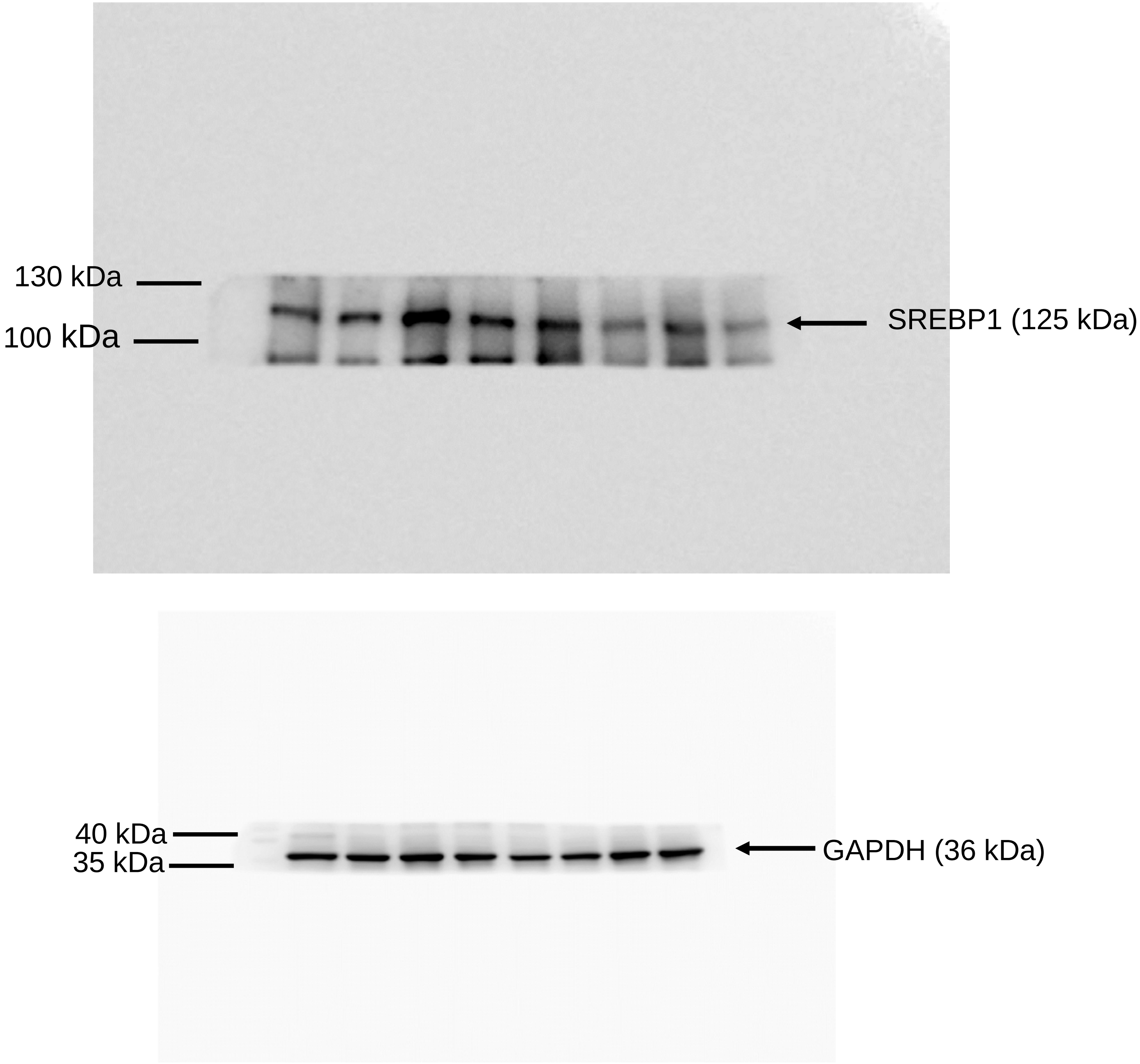

Figure 4c

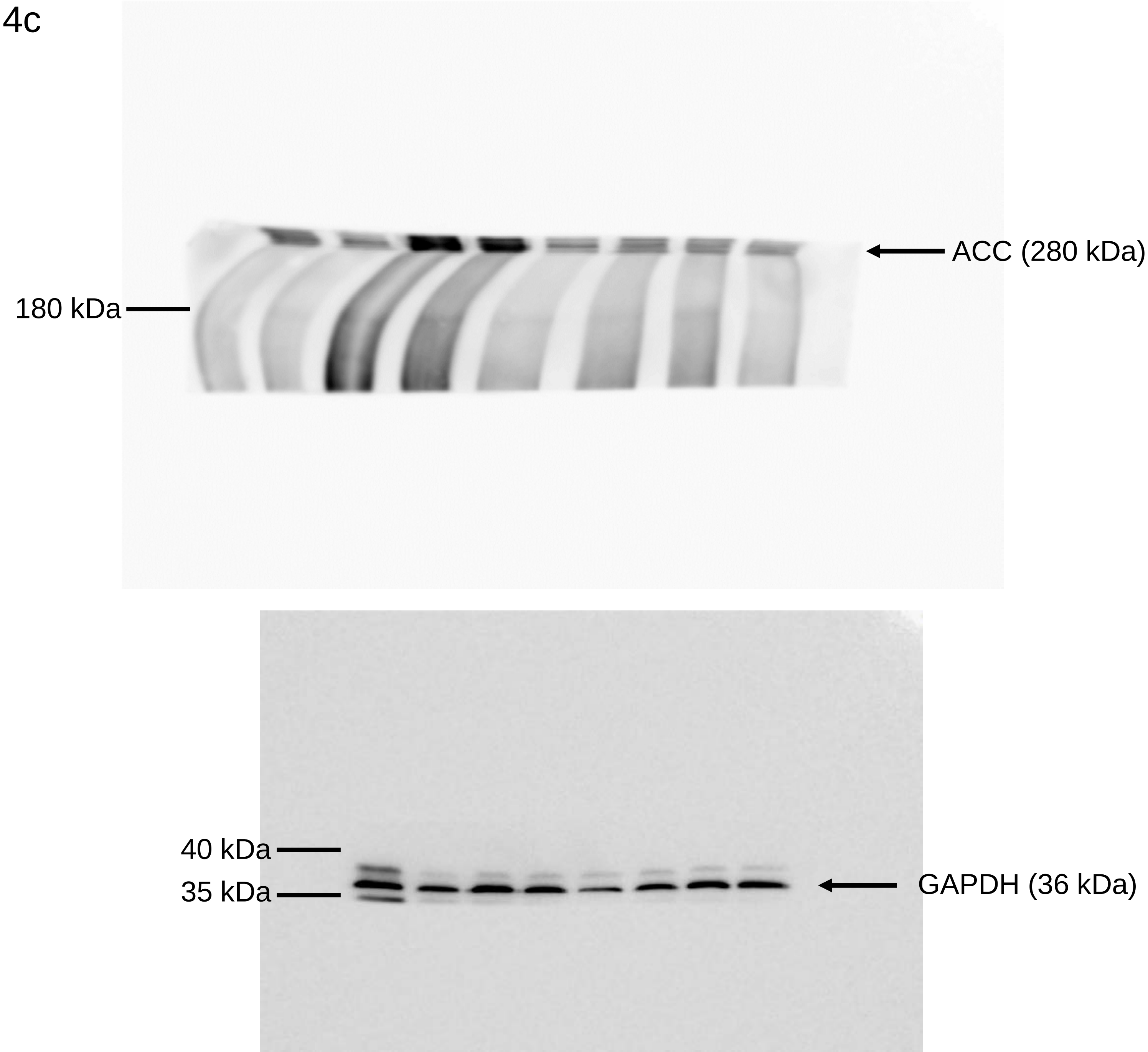

Figure 4c

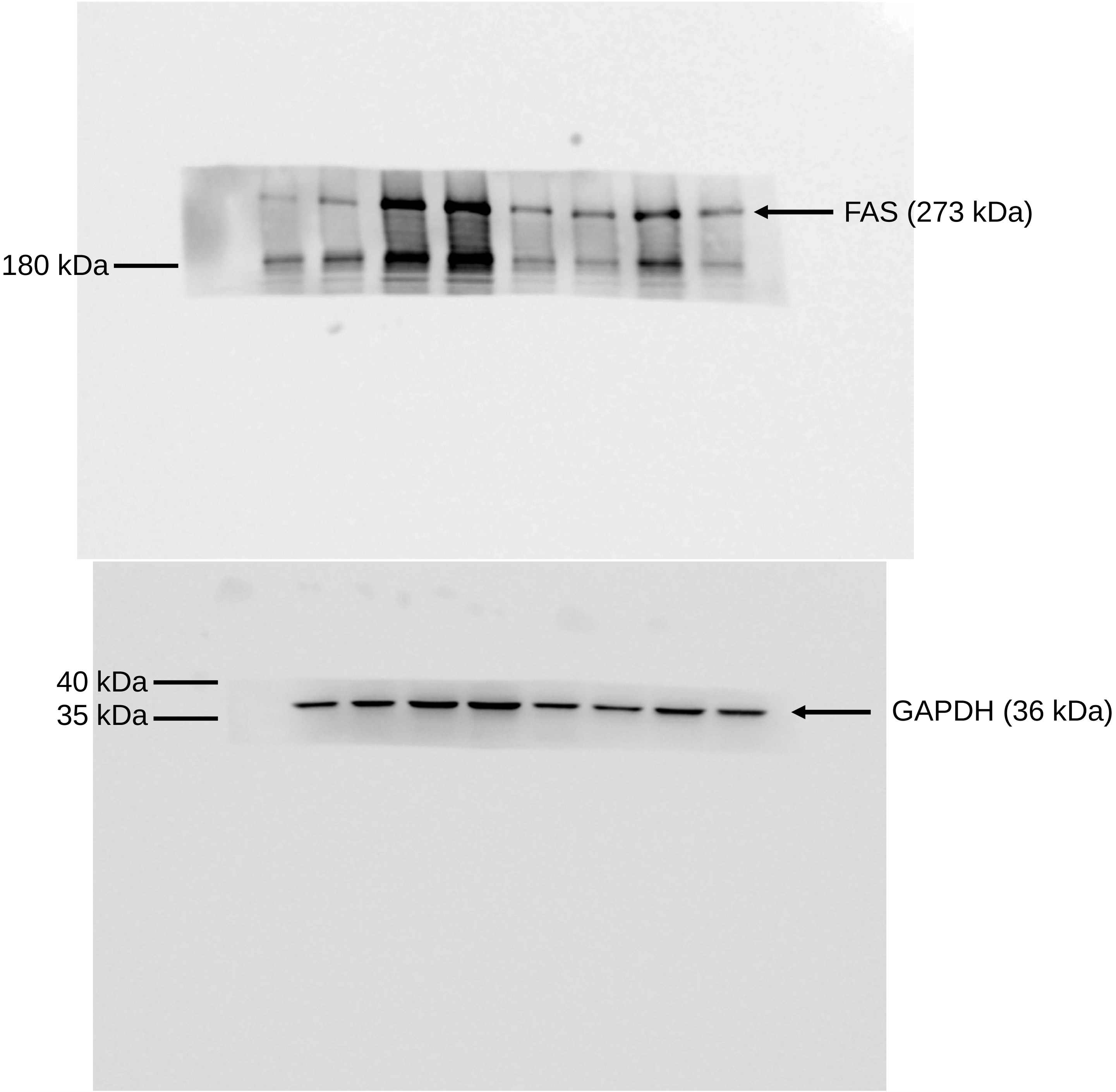

Figure 4c

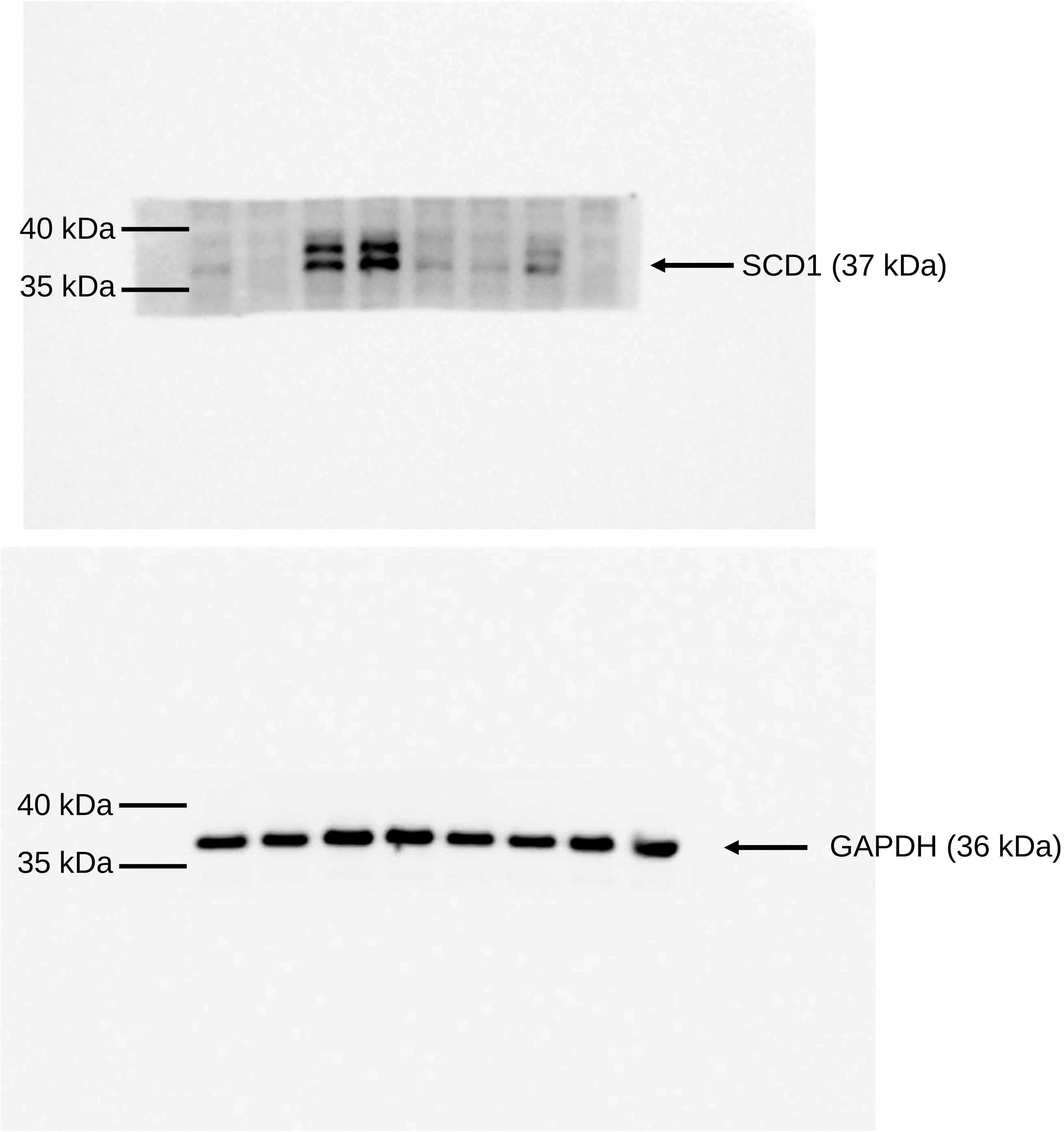

Figure 5a

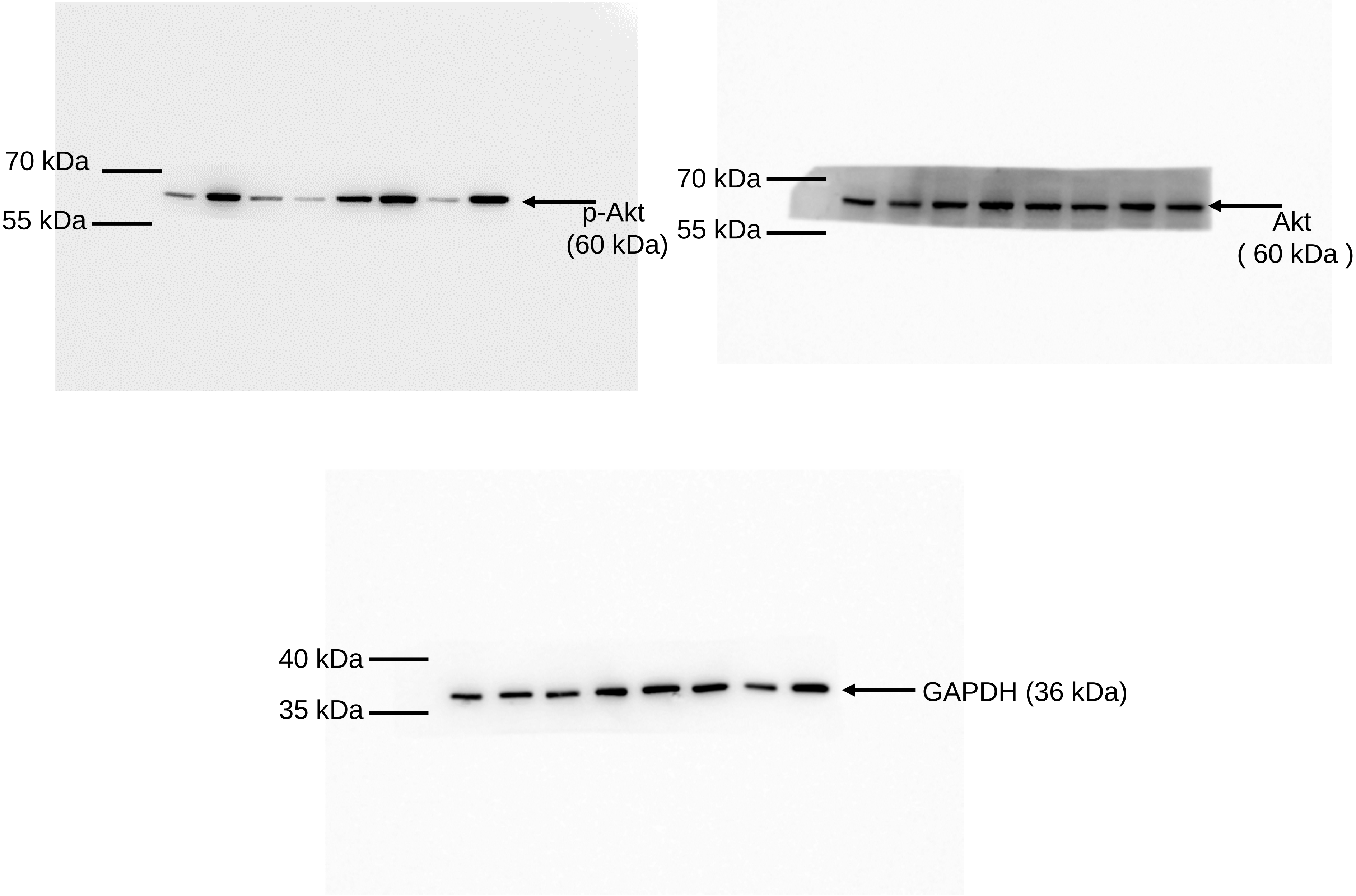
70 kDa
55 kDa
p-Akt
(60 kDa)
70 kDa
55 kDa
Akt
( 60 kDa )
40 kDa
35 kDa
GAPDH (36 kDa)

Figure 5b

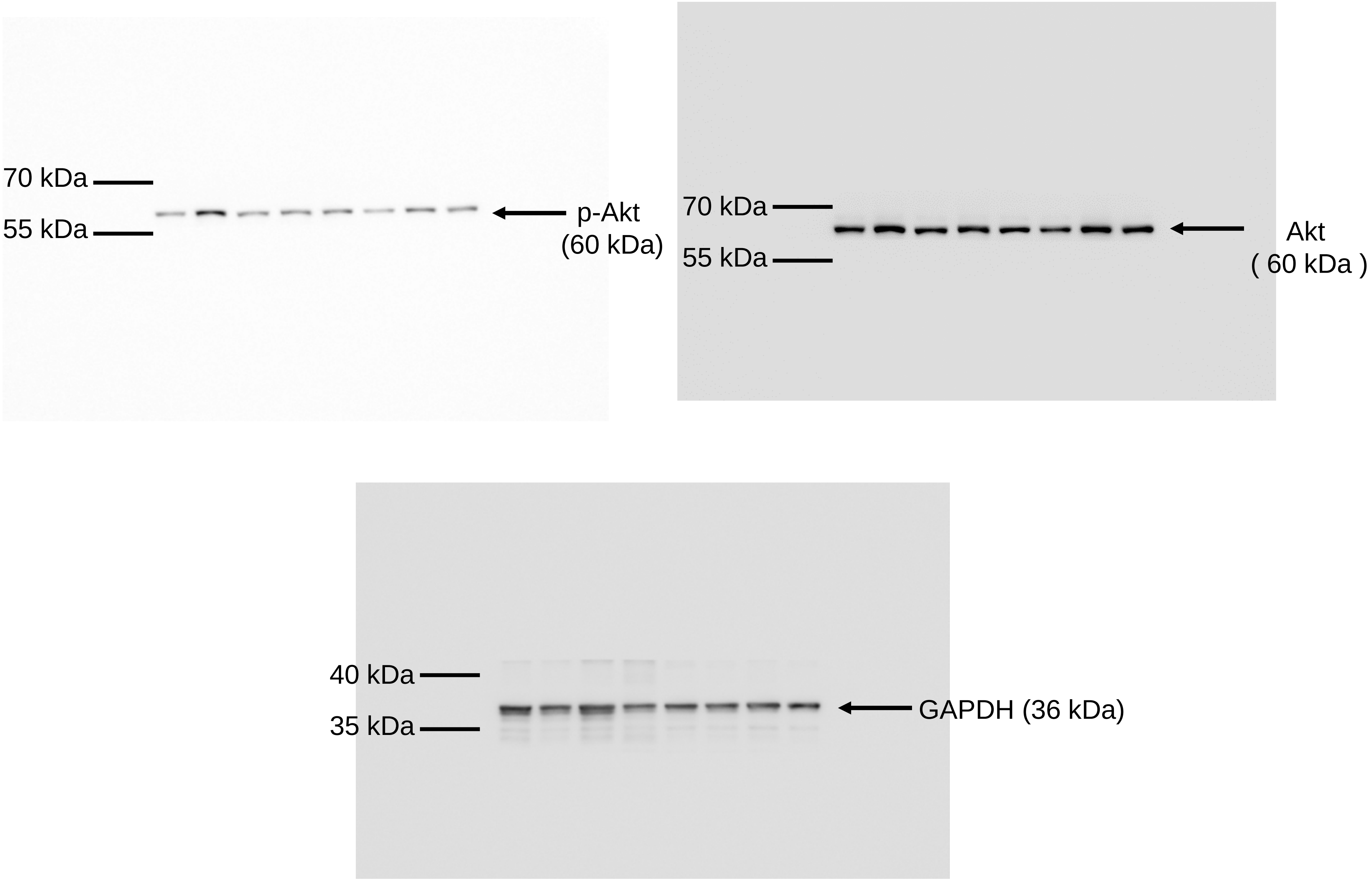
70 kDa
55 kDa
p-Akt
(60 kDa)
70 kDa
55 kDa
Akt
( 60 kDa )
40 kDa
35 kDa
GAPDH (36 kDa)

Figure 5c

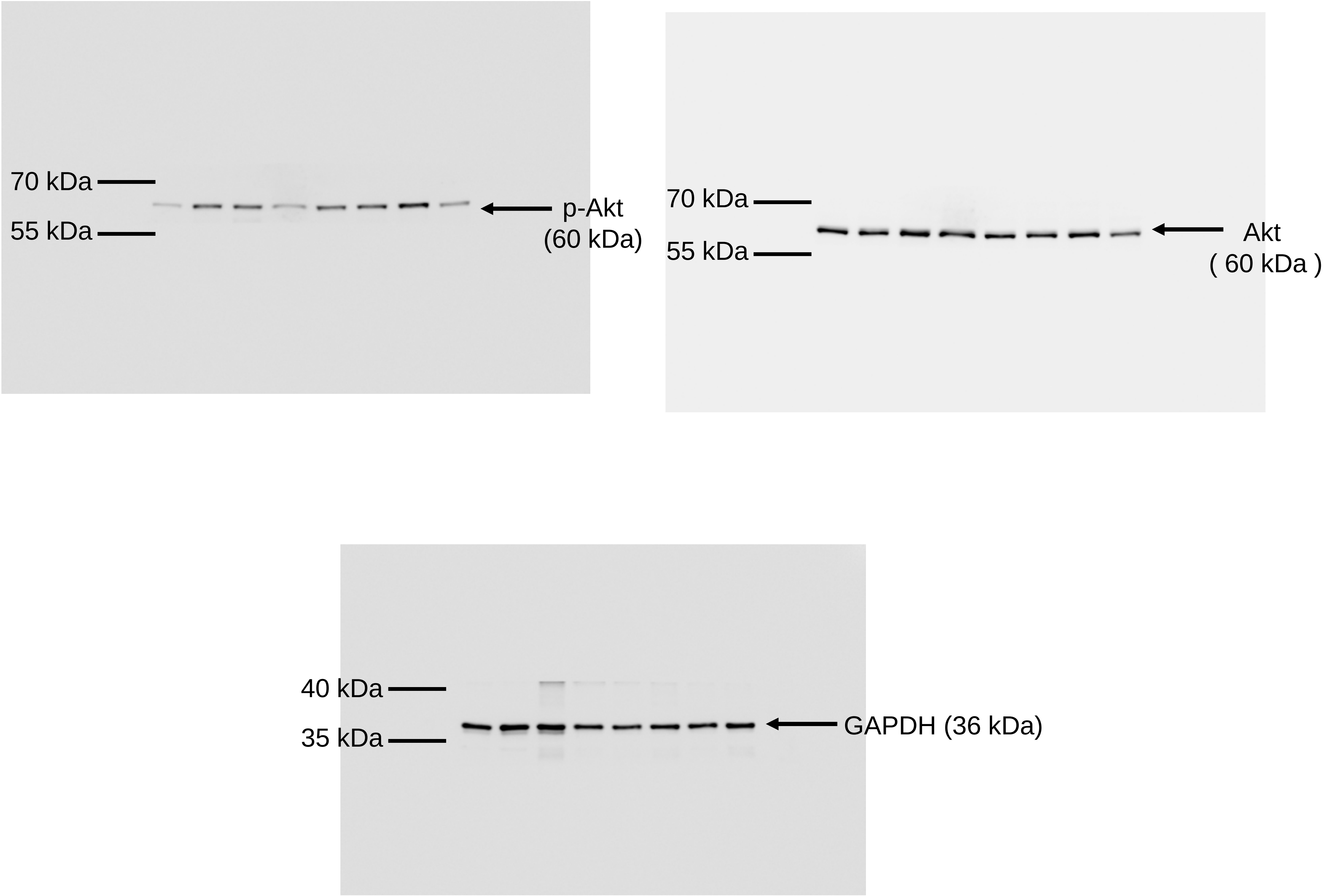
70 kDa
55 kDa
p-Akt
(60 kDa)
70 kDa
55 kDa
Akt
( 60 kDa )
40 kDa
35 kDa
GAPDH (36 kDa)

Figure 5d

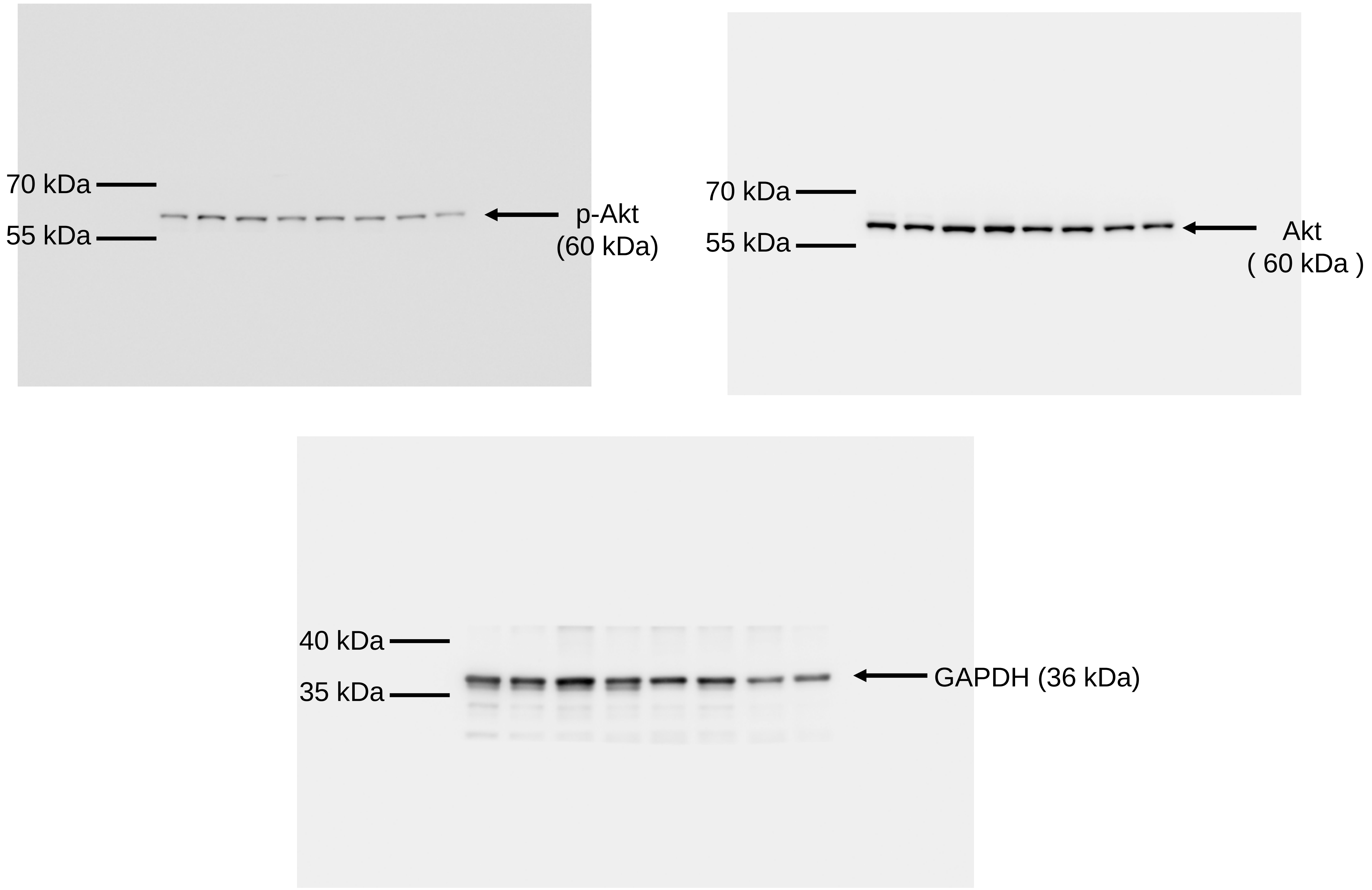
70 kDa
55 kDa
p-Akt
(60 kDa)
70 kDa
55 kDa
Akt
( 60 kDa )
40 kDa
35 kDa
GAPDH (36 kDa)

Figure 5e

shRNA#1

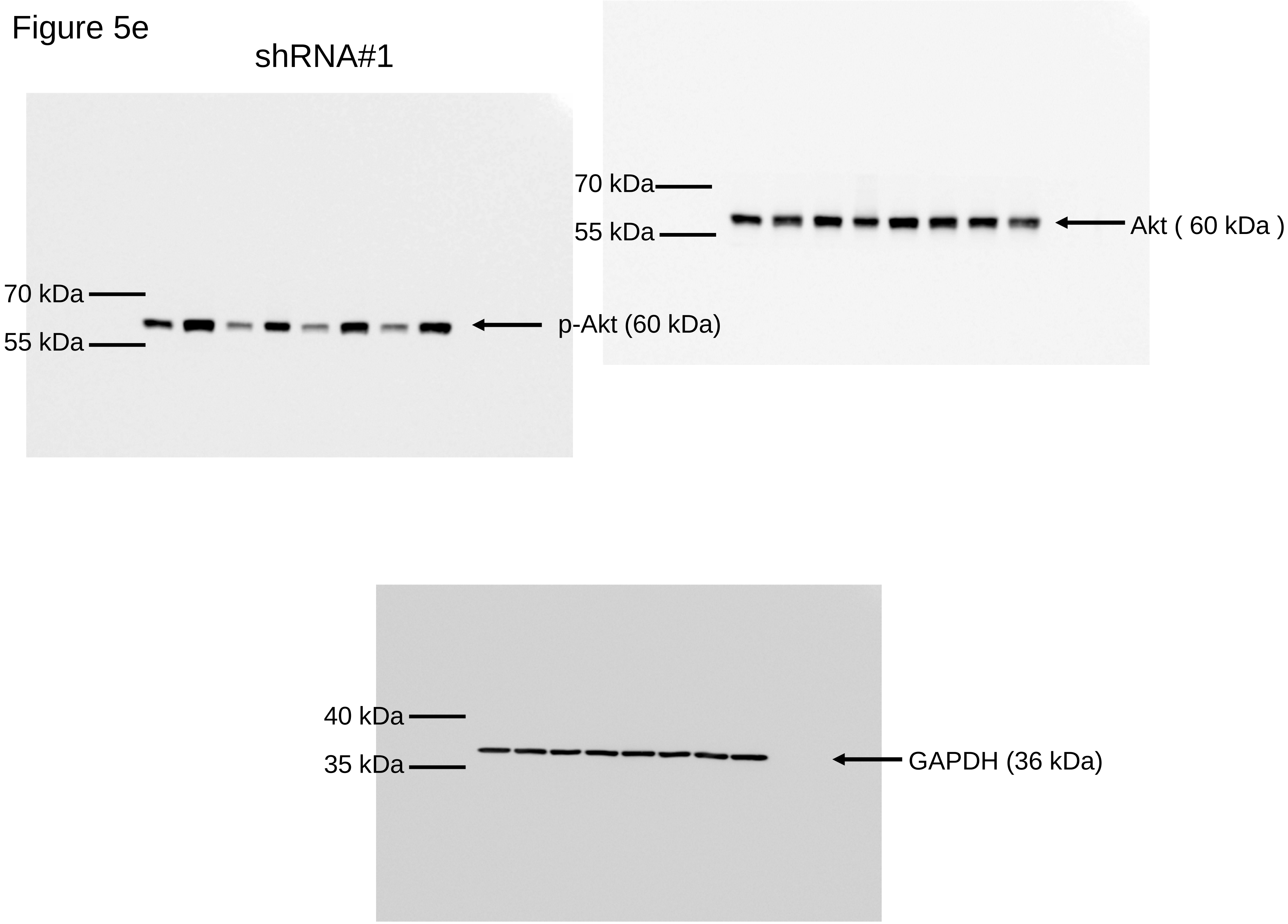

Figure 5e

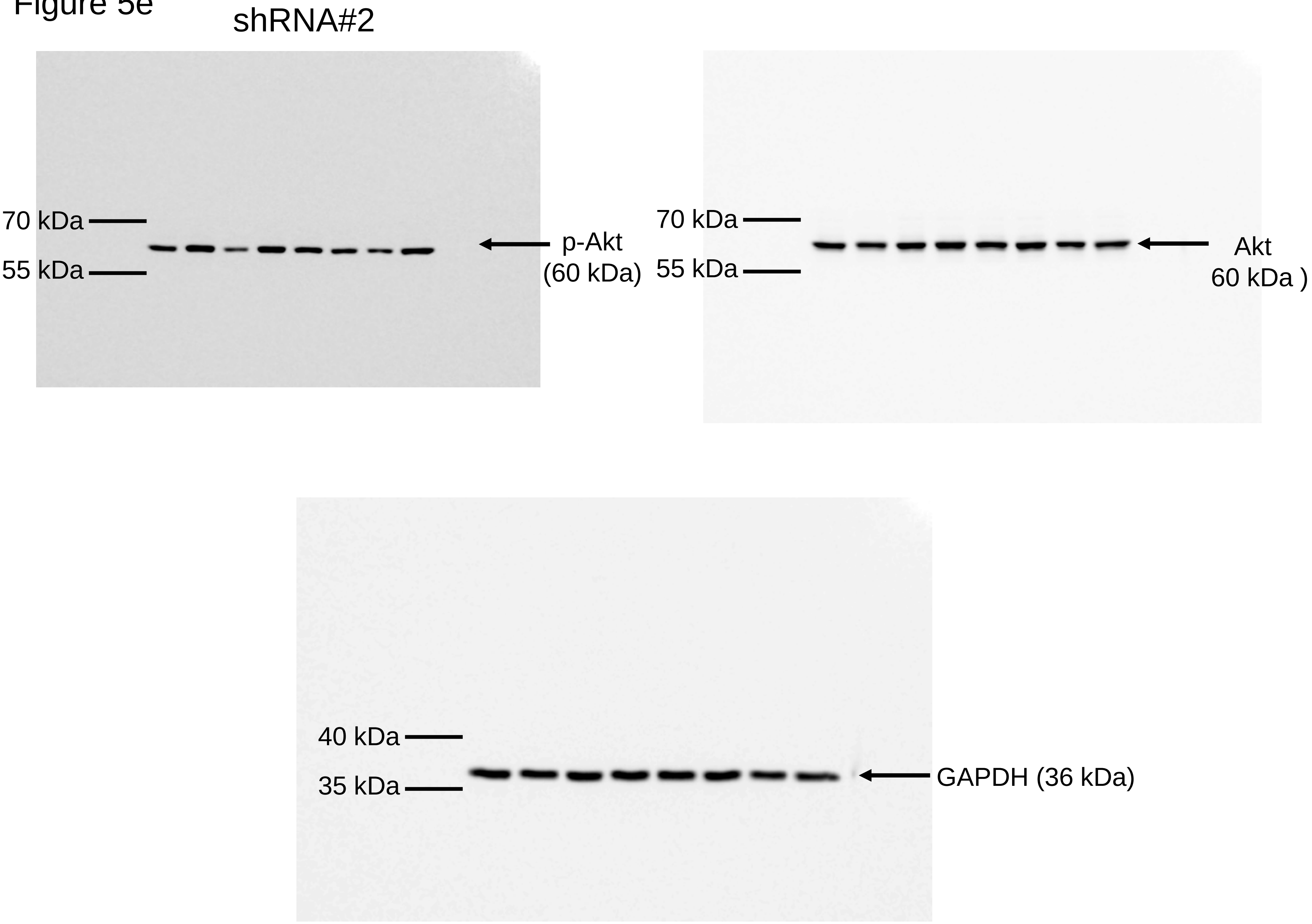
shRNA#2
70 kDa
55 kDa
p-Akt
(60 kDa)
70 kDa
55 kDa
Akt
60 kDa )
40 kDa
35 kDa
GAPDH (36 kDa)

Figure 5e

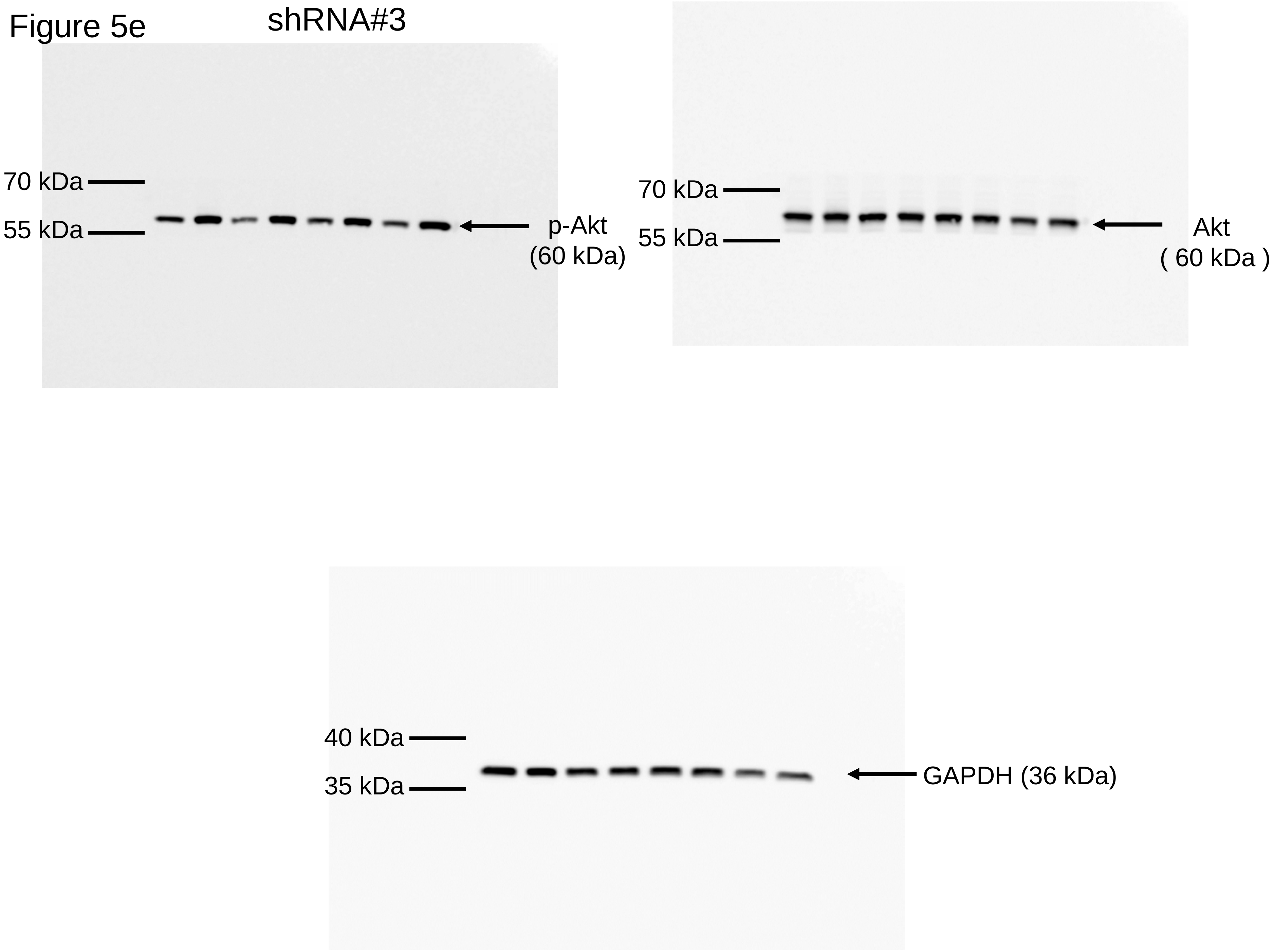
shRNA#3
70 kDa
55 kDa
p-Akt
(60 kDa)
70 kDa
55 kDa
Akt
( 60 kDa )
40 kDa
35 kDa
GAPDH (36 kDa)

Figure 6e

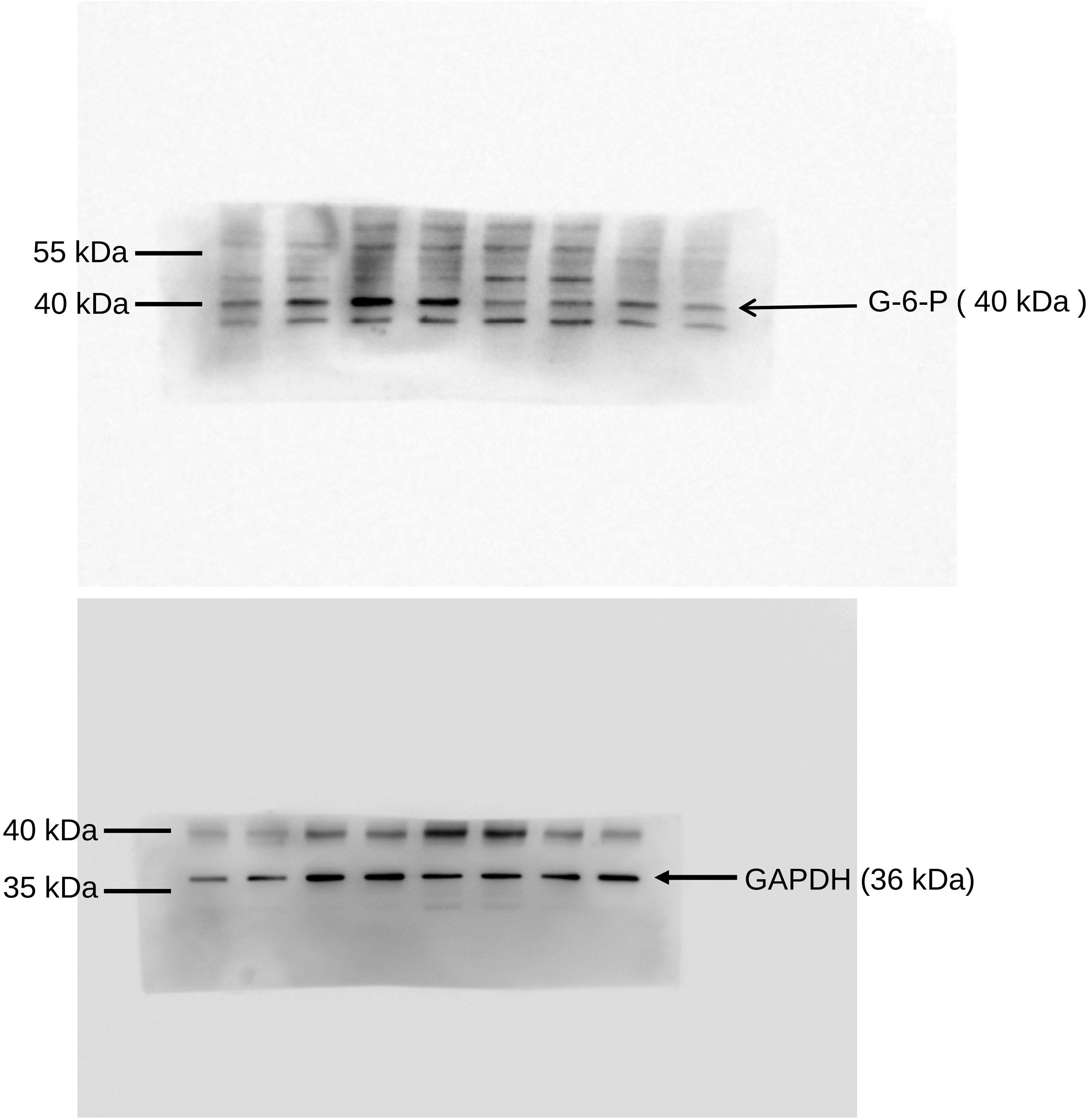

Figure 6e

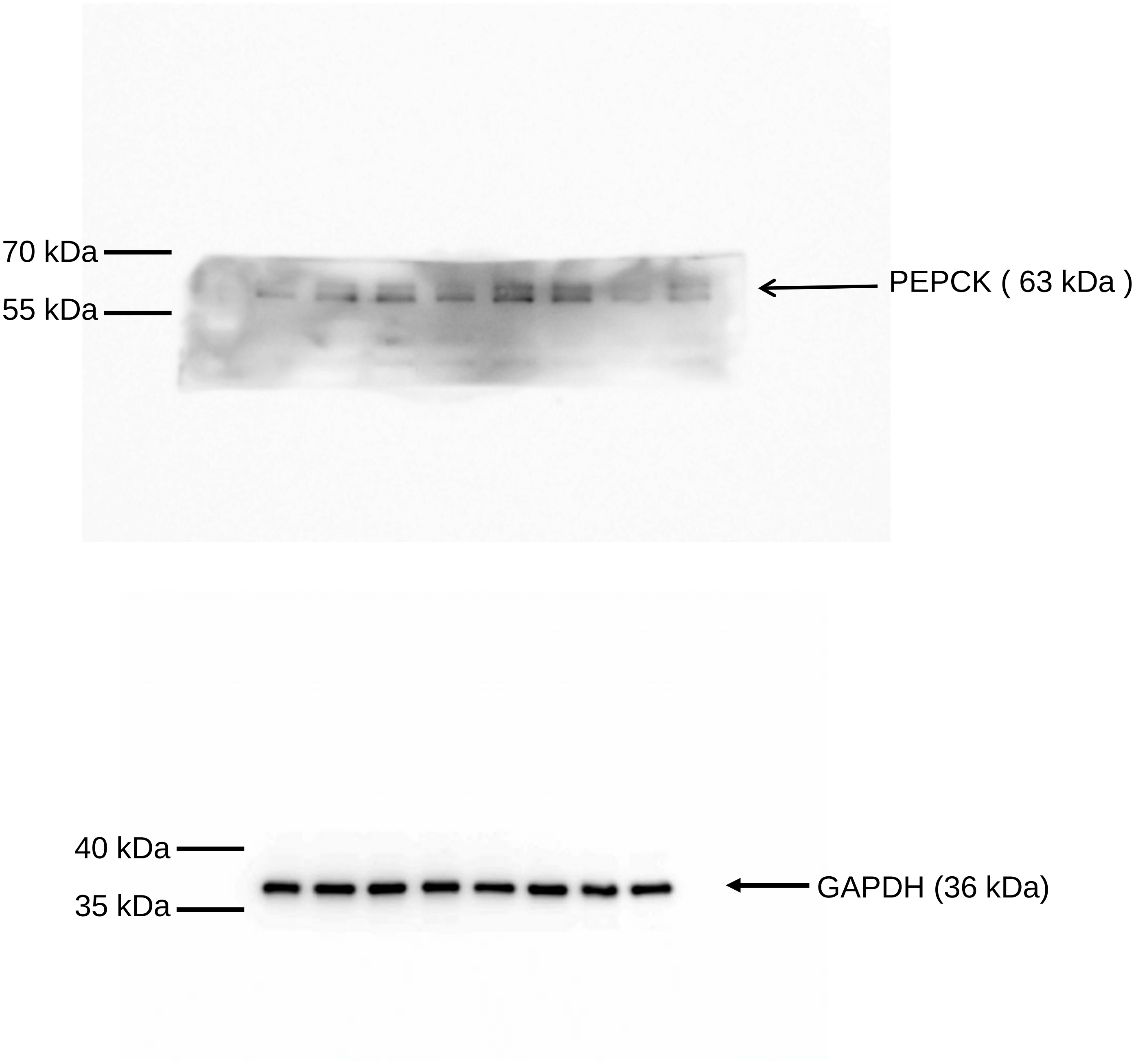

Figure 7d

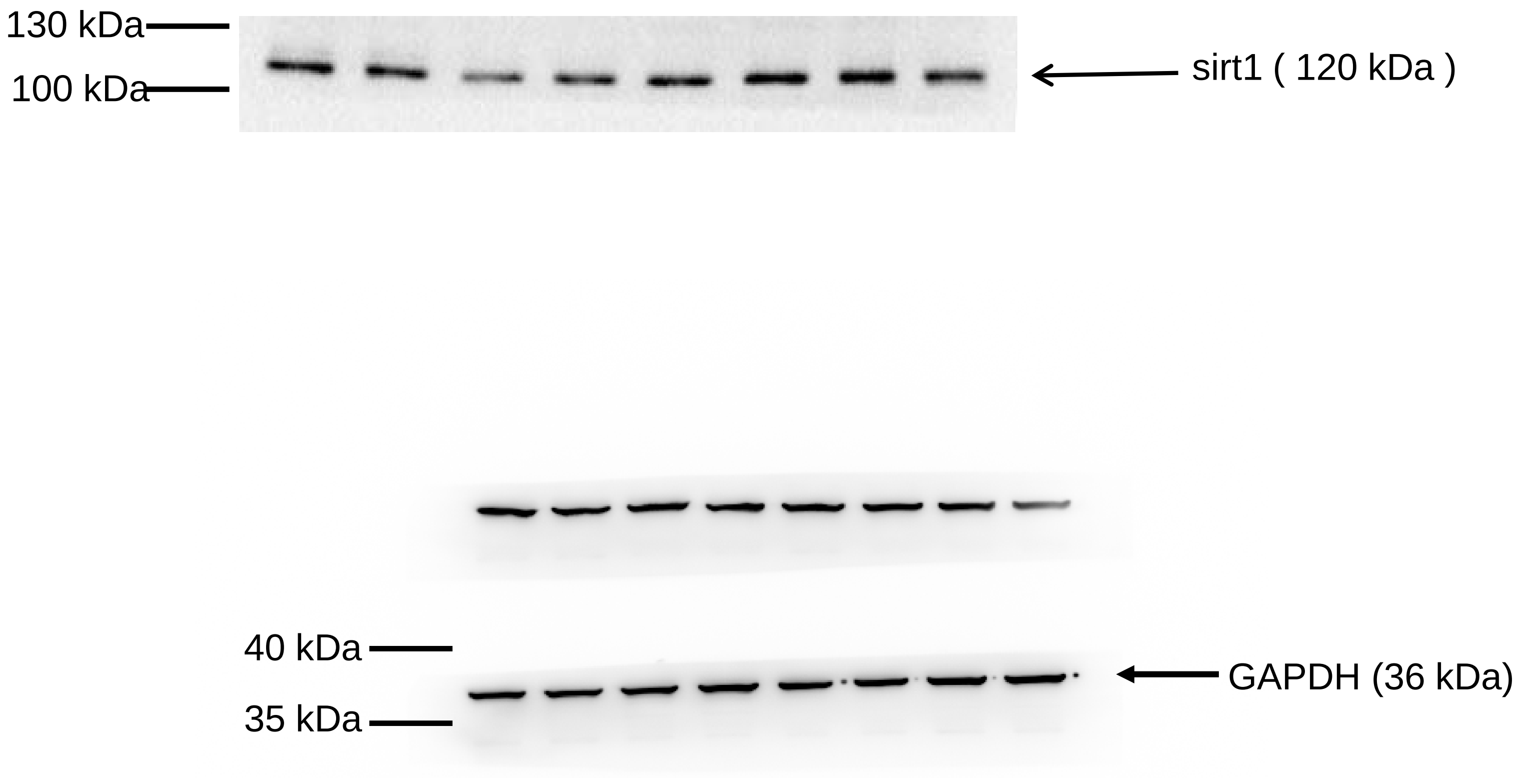

Figure 7d

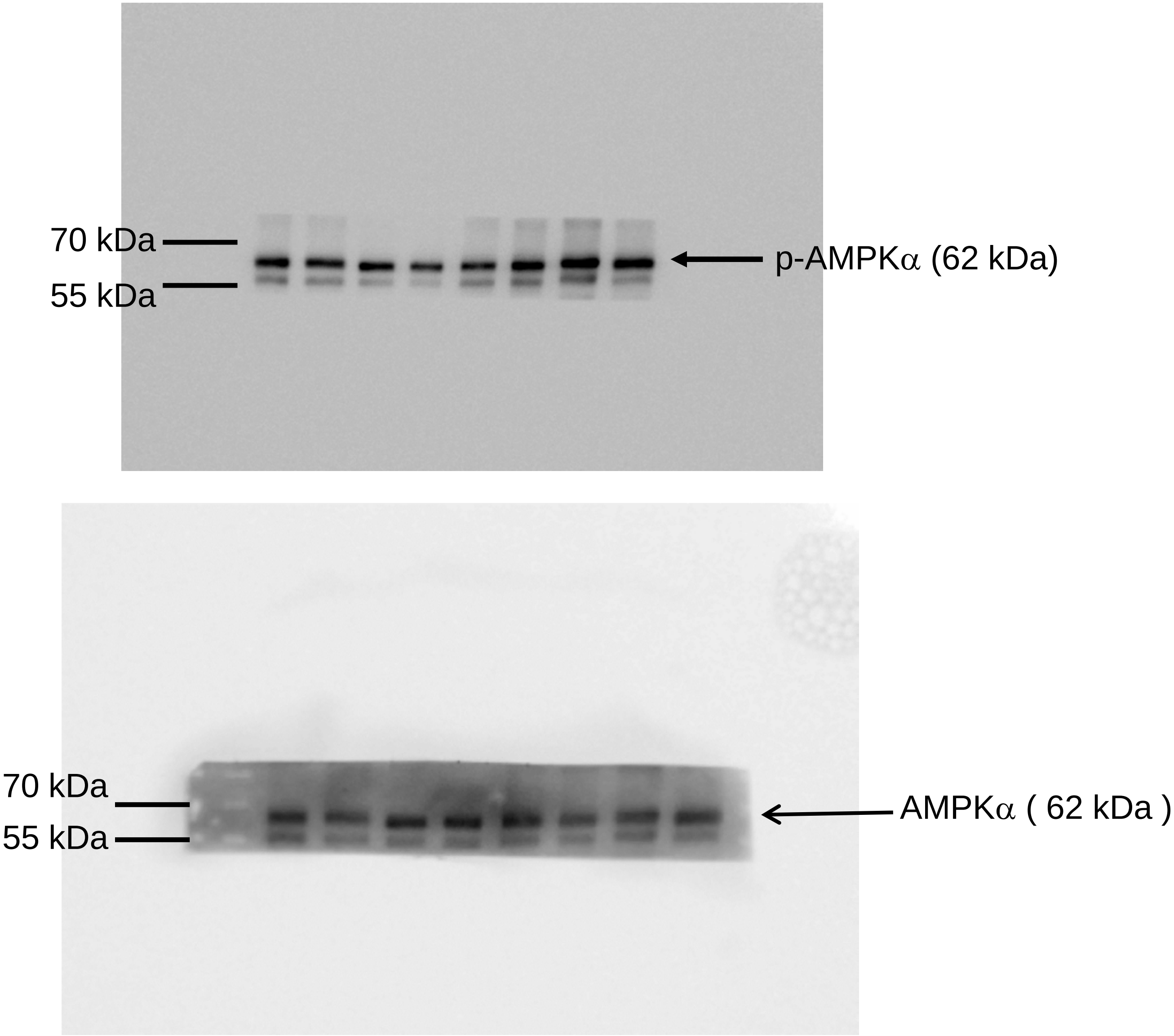

# Figure 7e

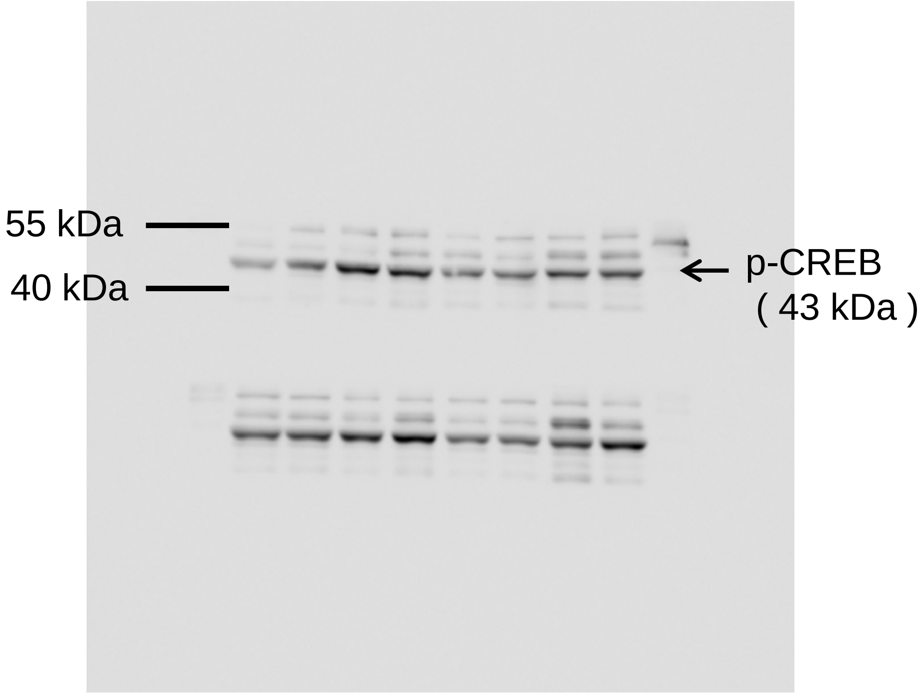

55 kDa
40 kDa
p-CREB
( 43 kDa )


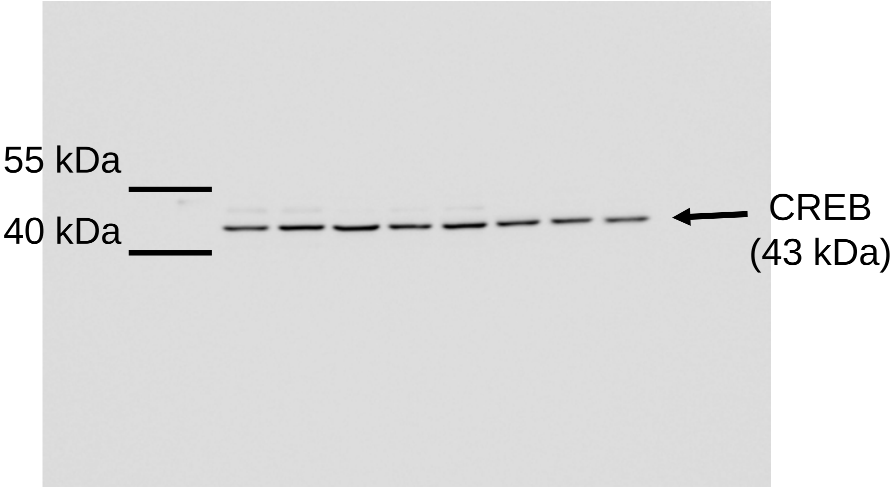

55 kDa
40 kDa
CREB
(43 kDa)


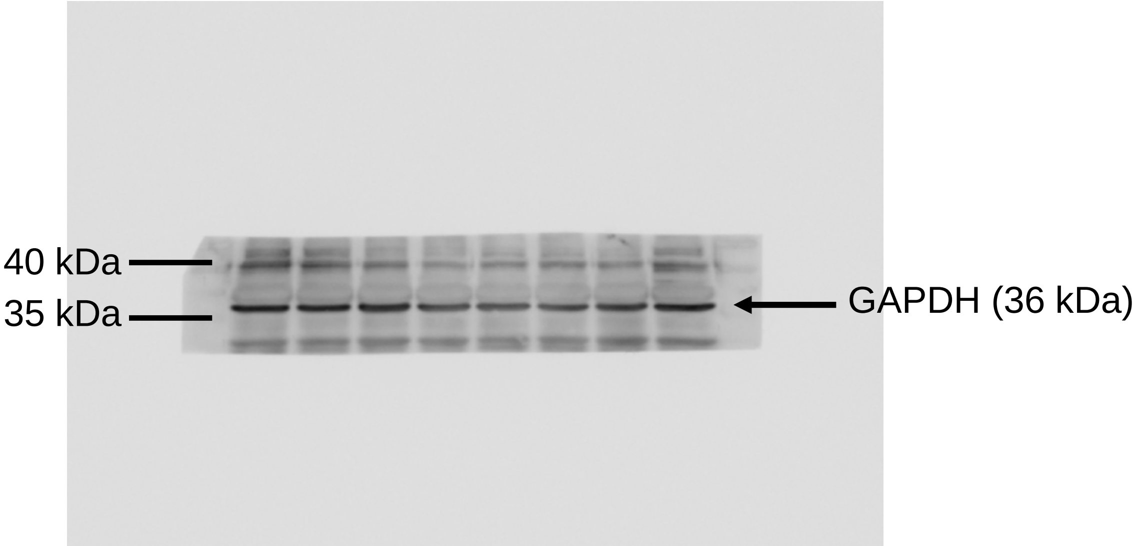

40 kDa
35 kDa
GAPDH (36 kDa)

Figure 7e

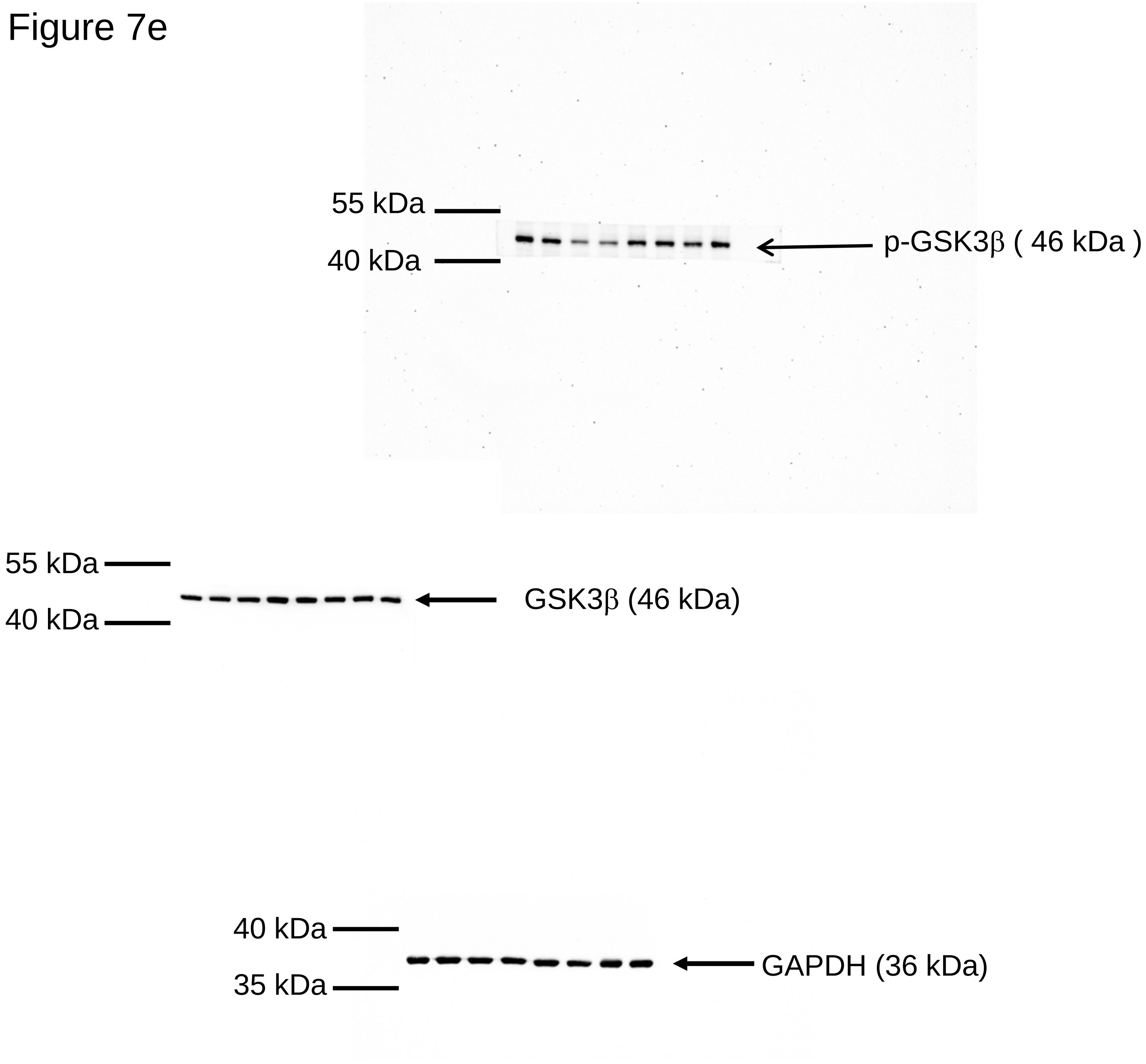
55 kDa
40 kDa
p-GSK3β ( 46 kDa )
55 kDa
40 kDa
GSK3β (46 kDa)
40 kDa
35 kDa
GAPDH (36 kDa)

Supplementary Figure 6c

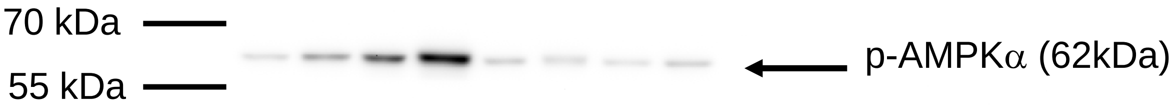


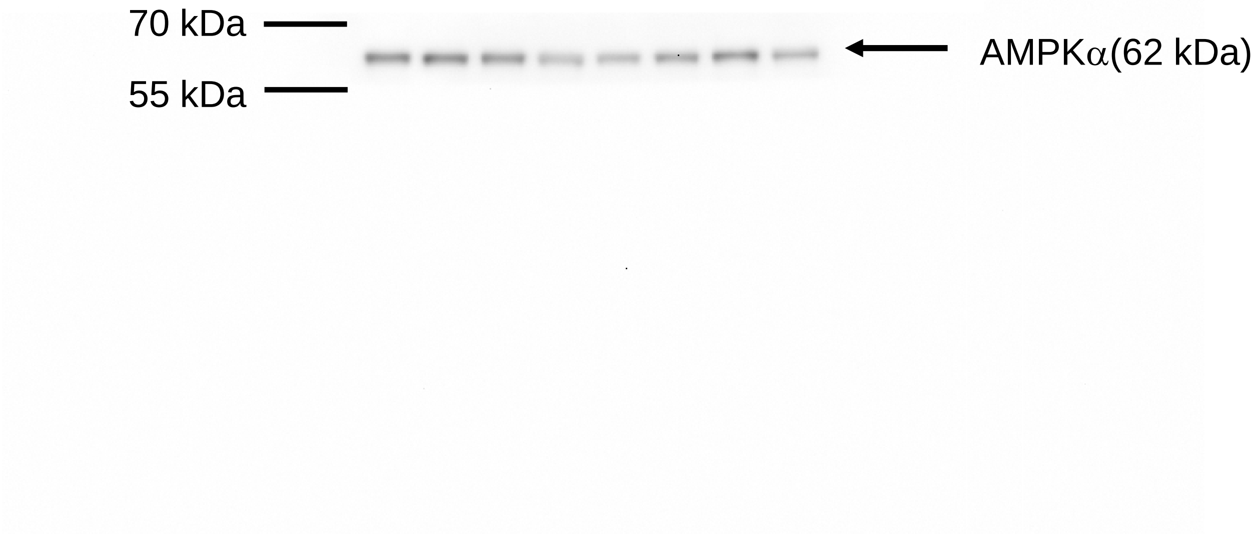

# Supplementary Figure 6c

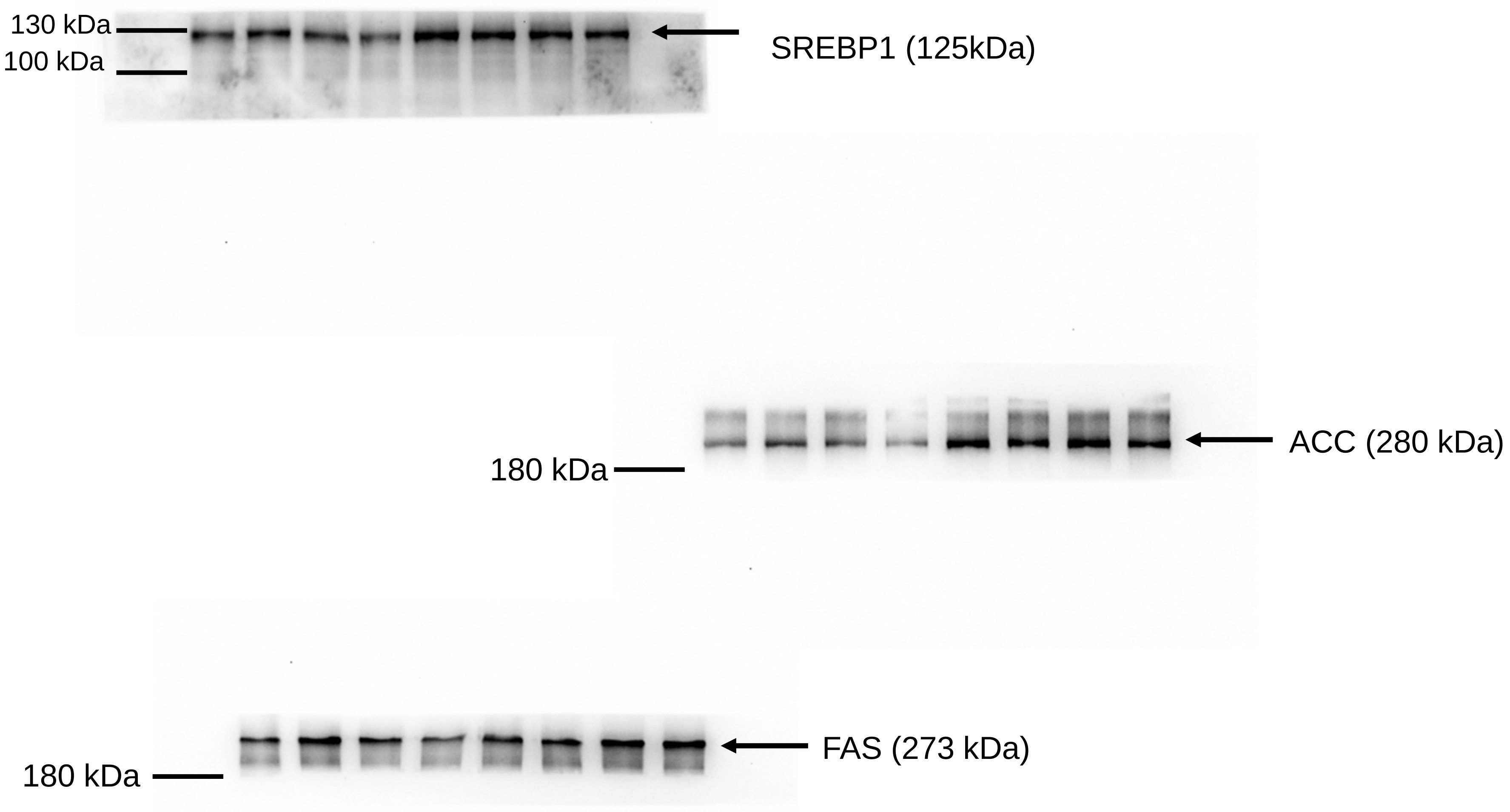

Supplementary Figure 6c

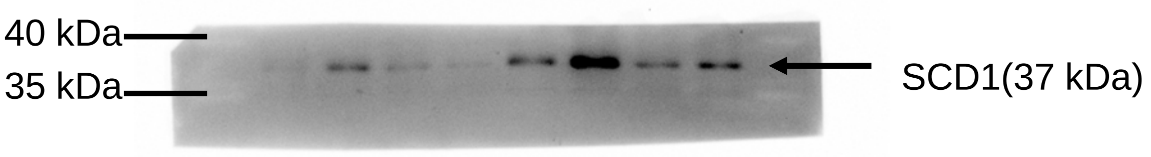


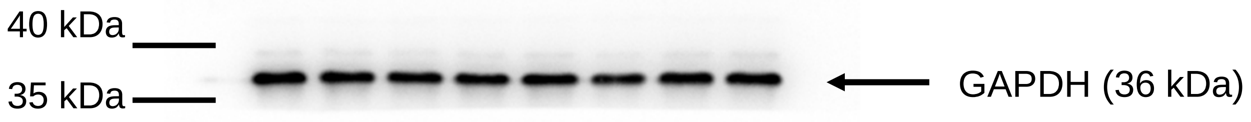

# Supplementary Figure 8

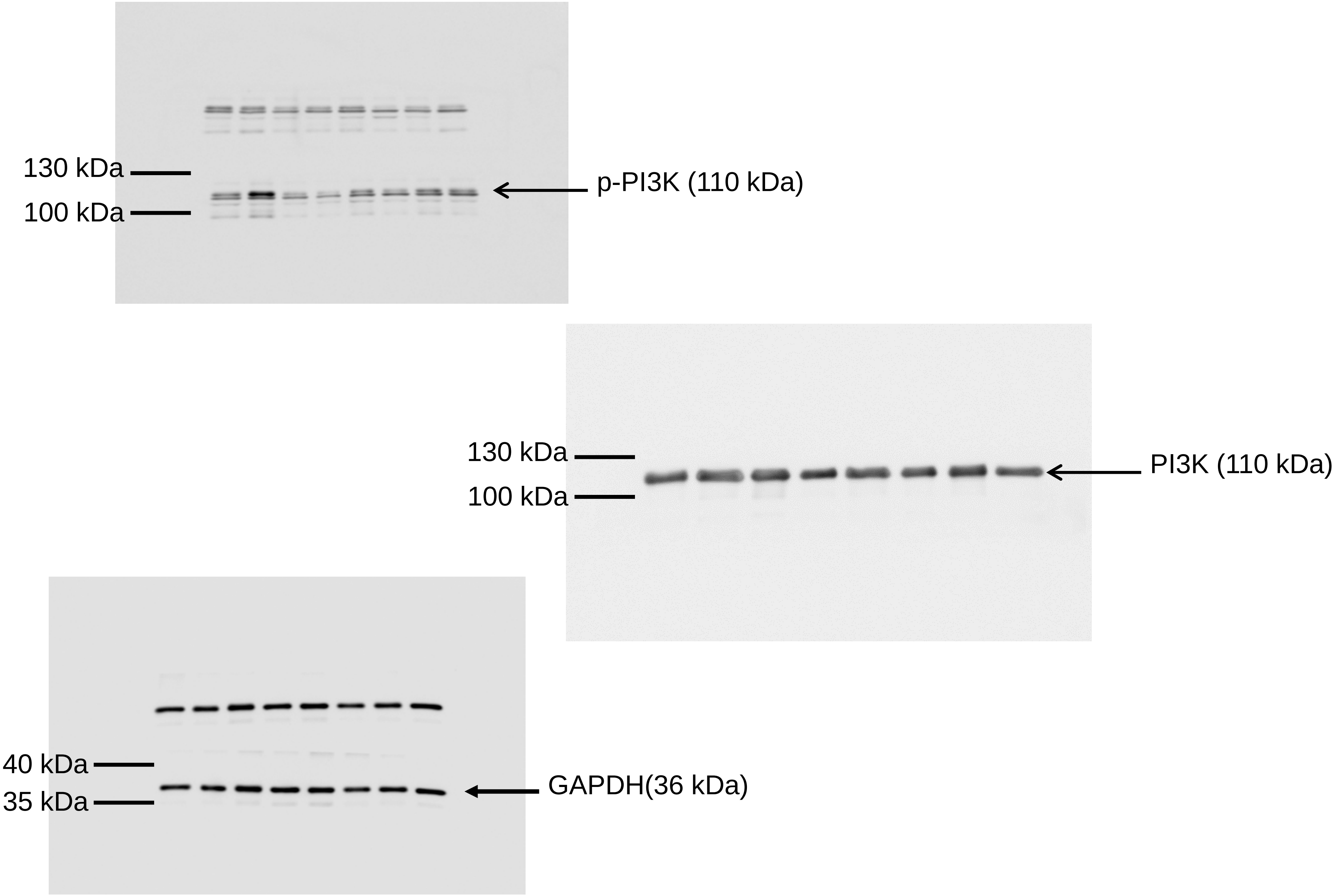

**Supplementary Table 1 – Name and sequences of primers for q-PCR.**

| Primer | Sequence |
|---|---|
| GCK | Forward 5'- GCA TCT CTG ACT TCC TGG ACA AG -3'<br>Reverse 5'- CTT GGT CCA GTT GAG CAG GAT G -3' |
| CD36 | Forward 5'-GAC TGG GAC CAT TGG TGA TGA-3'<br>Reverse 5'-AAG GCC ATC TCT ACC ATG CC-3' |
| PPARγ | Forward 5'-TTG ACA CCA TAC TTG AGC AGA-3'<br>Reverse 5'-CAG GAG CAG AGC AAA GAG GT-3' |
| PPARα | Forward 5'-GTT CAC CCT GAT TCC TGA TGT C-3'<br>Reverse 5'-CCT GCT TCC TGC CAC TTG-3' |
| PGC-1 | Forward 5'-TGC GGG ATG ATG GAG ACA-3'<br>Reverse 5'-GCG AAA GCG TCA CAG TA-3' |
| CPT1A | Forward 5'- ATC AAT CGG ACT CTG GAA ACG G-3'<br>Reverse 5'-TCA GGG AGT AGC GCA TGG T-3' |
| β-actin | Forward 5'-GGC CAA CCG TGA AAA GAT GA-3'<br>Reverse 5'-GAC CAG AGG CAT ACA GGG ACA-3' |
