# Supplementary material for: Deficiency of ADAR2 ameliorates metabolic-associated fatty liver disease via AMPK signaling pathways in obese mice
Source: Commun Biol. 2024 May 17;7:594. doi: 10.1038/s42003-024-06215-4 (PMC11101631; doi:10.1038/s42003-024-06215-4)
Supplement: Supplementary file 4 — Reporting Summary [file 42003_2024_6215_MOESM4_ESM.pdf]

nature portfolio

Corresponding author(s): Yun-Wen Chen
Last updated by author(s): Mar 10, 2024

Reporting Summary

Nature Portfolio wishes to improve the reproducibility of the work that we publish. This form provides structure for consistency and transparency in reporting. For further information on Nature Portfolio policies, see our Editorial Policies and the Editorial Policy Checklist.

Statistics

For all statistical analyses, confirm that the following items are present in the figure legend, table legend, main text, or Methods section.

n/a	Confirmed	
☐	☒	The exact sample size (*n*) for each experimental group/condition, given as a discrete number and unit of measurement
☐	☒	A statement on whether measurements were taken from distinct samples or whether the same sample was measured repeatedly
☐	☒	The statistical test(s) used AND whether they are one- or two-sided<br>*Only common tests should be described solely by name; describe more complex techniques in the Methods section.*
☒	☐	A description of all covariates tested
☐	☒	A description of any assumptions or corrections, such as tests of normality and adjustment for multiple comparisons
☐	☒	A full description of the statistical parameters including central tendency (e.g. means) or other basic estimates (e.g. regression coefficient) AND variation (e.g. standard deviation) or associated estimates of uncertainty (e.g. confidence intervals)
☐	☒	For null hypothesis testing, the test statistic (e.g. *F*, *t*, *r*) with confidence intervals, effect sizes, degrees of freedom and *P* value noted<br>*Give P values as exact values whenever suitable.*
☒	☐	For Bayesian analysis, information on the choice of priors and Markov chain Monte Carlo settings
☒	☐	For hierarchical and complex designs, identification of the appropriate level for tests and full reporting of outcomes
☒	☐	Estimates of effect sizes (e.g. Cohen's *d*, Pearson's *r*), indicating how they were calculated

Our web collection on statistics for biologists contains articles on many of the points above.

Software and code

Policy information about availability of computer code

Data collection	Images were observed under a bright field microscope. Bands in the immunoblots were quantified by using ImageQuant LAS 4000
Data analysis	All data were plotted and reported as mean±standard error of the mean (SEM). Significance was set at p < 0.05. Student's t test was adopted to analyze the data sets with a single factor (HFD effect). The body weight and energy intake of mice were analyzed using repeated measured two-way ANOVA. Ordinary two-way ANOVAs were used to analyze the results with two factors (HFD and ADAR2 KO). Sidak's post-hoc test was used to perform multiple comparison analysis after the two-way ANOVAs. Statistical analyses were performed with GraphPad Prism 8.0

For manuscripts utilizing custom algorithms or software that are central to the research but not yet described in published literature, software must be made available to editors and reviewers. We strongly encourage code deposition in a community repository (e.g. GitHub). See the Nature Portfolio guidelines for submitting code & software for further information.

Data

Policy information about availability of data

All manuscripts must include a data availability statement. This statement should provide the following information, where applicable:

- Accession codes, unique identifiers, or web links for publicly available datasets
- A description of any restrictions on data availability
- For clinical datasets or third party data, please ensure that the statement adheres to our policy

The data generated during the current study are available as a source data file and from the corresponding author on reasonable request.

Research involving human participants, their data, or biological material

Policy information about studies with human participants or human data. See also policy information about sex, gender (identity/presentation), and sexual orientation and race, ethnicity and racism.

Reporting on sex and gender	N/A
Reporting on race, ethnicity, or other socially relevant groupings	N/A
Population characteristics	N/A
Recruitment	N/A
Ethics oversight	N/A

Note that full information on the approval of the study protocol must also be provided in the manuscript.

Field-specific reporting

Please select the one below that is the best fit for your research. If you are not sure, read the appropriate sections before making your selection.

☒ Life sciences ☐ Behavioural & social sciences ☐ Ecological, evolutionary & environmental sciences

For a reference copy of the document with all sections, see nature.com/documents/nr-reporting-summary-flat.pdf

Life sciences study design

All studies must disclose on these points even when the disclosure is negative.

Sample size	Sample sizes were not predetermined based on statistical methods. Sample numbers were described in each figures for each genotypes. We chose p-value less than 0.05. The sample size for each experiment were commonly used in the field.
Data exclusions	No data was excluded
Replication	The main findings of the paper were confirmed by multiple complementary experiments. Results from current studies were consistently across multiple experiments. All similar replicates generating similar results.
Randomization	Same genotypes were randomly allocated to experimental groups
Blinding	Not blind to experimenters. However, different experiments were performed by different experimenters and the conclusion of results are supporting each other. Instead, we relied upon independent experimenter analysis and similar experiment to confirm the results.

Reporting for specific materials, systems and methods

We require information from authors about some types of materials, experimental systems and methods used in many studies. Here, indicate whether each material, system or method listed is relevant to your study. If you are not sure if a list item applies to your research, read the appropriate section before selecting a response.

Materials & experimental systems

n/a	Involved in the study	
☐	☒	Antibodies
☐	☒	Eukaryotic cell lines
☒	☐	Palaeontology and archaeology
☐	☒	Animals and other organisms
☒	☐	Clinical data
☒	☐	Dual use research of concern
☒	☐	Plants

Methods

n/a	Involved in the study	
☒	☐	ChIP-seq
☒	☐	Flow cytometry
☒	☐	MRI-based neuroimaging

Antibodies

Antibodies used	ACC (Cat. #: 3676), AKT (Cat. #: 9272), phospho-AKT (Cat. #: 4060), AMPKa (Cat. #: 5831), phospho-AMPKa (Cat. #: 2535), CREB (Cat. #: 9197), phospho-CREB (Cat. #: 9198), GSK3β (Cat. #: 5676), phospho-GSK3β (Cat. #: 9327), FAS (Cat. #: 3180), PEPCK (Cat. #: 12940),

SCD1 (Cat. #: 2794), sirt1 (Cat. #: 8469) were from Cell Signaling Technology. Antibodies against G-6-P (Cat. #: ab83690) were from Abcam. Antibodies against GAPDH (Cat. #: MA5-15738) were from Thermo Fisher. Antibodies against SREBP-1(Cat. #: sc-13551) were from Santa Cruz.

Validation	ACC (Cat. #: 3676), AKT (Cat. #: 9272), phospho-AKT (Cat. #: 4060), AMPKa (Cat. #: 5831), phospho-AMPKa (Cat. #: 2535), CREB (Cat. #: 9197), phospho-CREB (Cat. #: 9198), GSK3β (Cat. #: 5676), phospho-GSK3β (Cat. #: 9327), FAS (Cat. #: 3180), PEPCK (Cat. #: 12940), SCD1 (Cat. #: 2794), sirt1 (Cat. #: 8469) were from Cell Signaling Technology. Antibodies against G-6-P (Cat. #: ab83690) were from Abcam. Antibodies against GAPDH (Cat. #: MA5-15738) were from Thermo Fisher. Antibodies against SREBP-1(Cat. #: sc-13551) were from Santa Cruz.

Eukaryotic cell lines

Policy information about cell lines and Sex and Gender in Research

Cell line source(s)	Huh7 cells
Authentication	none of the cell lines used were authentication
Mycoplasma contamination	all cell lines tested negative for mycoplasma contamination
Commonly misidentified lines (See ICLAC register)	N/A

Animals and other research organisms

Policy information about studies involving animals; ARRIVE guidelines recommended for reporting animal research, and Sex and Gender in Research

Laboratory animals	mouse (B6129S)
Wild animals	We did not use wild animals
Reporting on sex	we focused on male mice for the rest of the studies.
Field-collected samples	Did not involve collection in the field
Ethics oversight	All experimental procedures of animal studies were approved by the Institutional Animal Care and Use Committees (IACUC) of National Cheng Kung University (IACUC approval number: 106069).

Note that full information on the approval of the study protocol must also be provided in the manuscript.

Plants

Seed stocks	N/A
Novel plant genotypes	N/A
Authentication	N/A
